# Evaluation of Spatial Pattern of Altered Flow Regimes on a River Network Using a Distributed Hydrological Model

**DOI:** 10.1371/journal.pone.0133833

**Published:** 2015-07-24

**Authors:** Masahiro Ryo, Yuichi Iwasaki, Chihiro Yoshimura, Oliver C. Saavedra V.

**Affiliations:** 1 Department of Civil Engineering, Tokyo Institute of Technology, Meguro-ku, Tokyo, Japan; 2 Department of Fish, Wildlife, and Conservation Biology, Colorado State University, Fort Collins, Colorado, United States of America; Tianjin University, CHINA

## Abstract

Alteration of the spatial variability of natural flow regimes has been less studied than that of the temporal variability, despite its ecological importance for river ecosystems. Here, we aimed to quantify the spatial patterns of flow regime alterations along a river network in the Sagami River, Japan, by estimating river discharge under natural and altered flow conditions. We used a distributed hydrological model, which simulates hydrological processes spatiotemporally, to estimate 20-year daily river discharge along the river network. Then, 33 hydrologic indices (i.e., Indicators of Hydrologic Alteration) were calculated from the simulated discharge to estimate the spatial patterns of their alterations. Some hydrologic indices were relatively well estimated such as the magnitude and timing of maximum flows, monthly median flows, and the frequency of low and high flow pulses. The accuracy was evaluated with correlation analysis (r > 0.4) and the Kolmogorov–Smirnov test (α = 0.05) by comparing these indices calculated from both observed and simulated discharge. The spatial patterns of the flow regime alterations varied depending on the hydrologic indices. For example, both the median flow in August and the frequency of high flow pulses were reduced by the maximum of approximately 70%, but these strongest alterations were detected at different locations (i.e., on the mainstream and the tributary, respectively). These results are likely caused by different operational purposes of multiple water control facilities. The results imply that the evaluation only at discharge gauges is insufficient to capture the alteration of the flow regime. Our findings clearly emphasize the importance of evaluating the spatial pattern of flow regime alteration on a river network where its discharge is affected by multiple water control facilities.

## Introduction

Freshwater biodiversity is decreasing more rapidly than in terrestrial or marine waters [1, 2]. The alteration of natural flow regimes owing to water control facilities, such as dams [3–5], is responsible for this decrease, as well as water pollution and overexploitation [6]. Rapid and severe alteration of river discharge variability degrades ecosystems because aquatic species evolve responding to natural flow regimes and thus may fail to adapt to such changes [7]. Impacts of flow alterations on freshwater ecosystems (e.g., loss of species and decline of biomass) were reported for a variety of taxonomic groups: macroinvertebrates, fish, and vegetation [8, 9]. Therefore, assessing the effect of flow regime modification is essential for the conservation of freshwater ecosystems.

In a watershed, natural flow regimes are spatially heterogeneous because of the distribution of precipitation, topology, channel morphology, landuse cover, and soil properties [10]. In general, the spatial variability of environmental factors, including the flow regime along a river network, shapes biological diversity and productivity [11]. For instance, tributaries with variable discharge characteristics increase habitat heterogeneity, possibly shaping fish distributions along a river network [12]. Hence, the spatial heterogeneity of the flow regime would play an important role in maintaining biodiversity in a watershed. However, the flow regime is often altered by water control facilities with different operational purposes at multiple locations on a river network. Thus, for precise understanding of their cumulative impacts on flow regime, these alterations should be spatially evaluated (e.g., along the entire river network) [13]. By evaluating the spatial pattern of flow alterations, we can identify sections along a river network where the flow regime is critically affected. Such information is valuable to river management.

However, despite the importance of spatial variability in flow regimes, the evaluation of flow regime alterations has been typically conducted based on single or few representative locations per river [14, 15]. The spatial pattern of altered flow regimes has been seldom assessed owing to the limited distribution of discharge gauges. For spatial evaluation, estimates along the river network are needed, including sections where observations of the river discharge are unavailable. In this regard, statistical and hydrological (rainfall–runoff) modeling approaches are promising [16]. Distributed hydrological models (DHMs), which describe the spatiotemporal heterogeneity of dominant hydrological processes in a catchment area, can simulate discharge at any point along a river network [17]. A notable advantage of DHMs is the simulation flexibility when making predictions based on multiple scenarios, whereas statistical models are solely developed based on empirical correlations in the available data (see comparative discussion in [18]). Despite these appealing features, DHMs have been rarely used to assess the spatial pattern of altered flow regimes compared with statistical models [14, 19–21].

The use of simulated discharge is becoming popular for prediction of ecological responses [16]. However, to maximize the utility of the DHMs, it is essential to pay attention to the accuracy of the simulation. If the simulation accuracy is low, its use would result in unreliable prediction and may lead to erroneous implications for management [18]. In this regard, identifying the general limitations of hydrological models for simulating various flow characteristics is particularly important. However, few studies have addressed the issue (but see [22]) and more studies in different areas and conditions are required.

**Fig 1:**
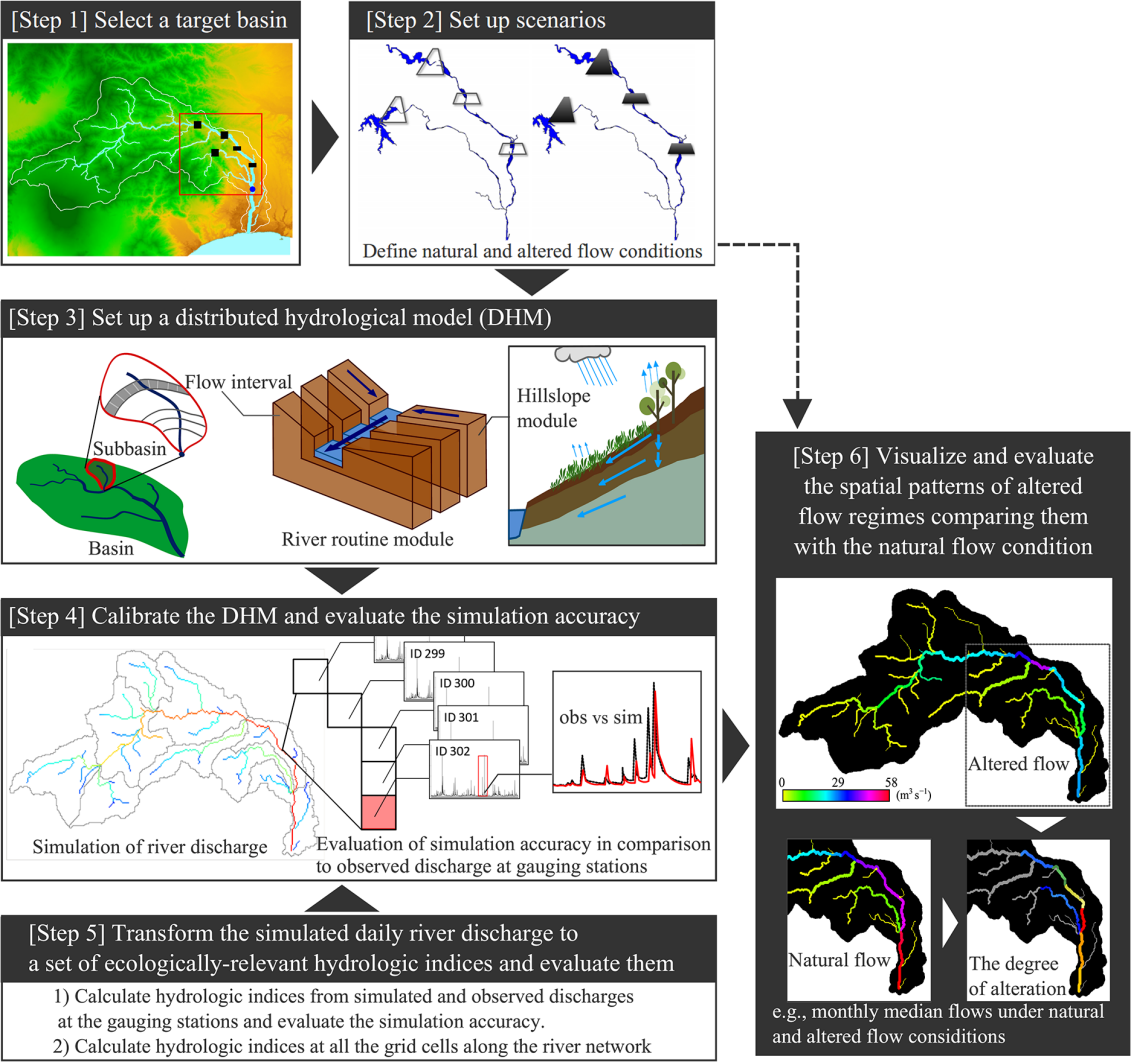
The framework for evaluating the spatial pattern of flow regimes using a hydrological model.

In this study, we aimed to evaluate the spatial patterns of flow regime alterations in a river basin where water control facilities modify river discharge. We applied the framework shown in Fig 1 to the Sagami River in Japan. A DHM simulates daily river discharges along the river network under natural and altered flow conditions after calibration and validation. A set of hydrologic indices were calculated from the simulated daily discharges under both flow conditions to evaluate various discharge characteristics. The accuracy of the hydrologic indices calculated from the simulated discharge was evaluated at the gauging stations based on a correlation analysis and a Kolmogorov–Smirnov test. Then, reasonably simulated hydrologic indices were further used to estimate and illustrate the spatial patterns of the altered flow regimes. To our knowledge, no study has applied DHMs for evaluating spatial patterns of various flow characteristics while considering accuracy. In addition, we discuss the reliability and limitation of such DHM application to estimate flow regimes.

## Method

The framework (Fig 1) was applied to evaluate the degree of flow regime alteration on the stream network based on scenario analysis. Such an evaluation is performed after assessing the reliability of the estimated spatial patterns of flow regimes using various hydrologic indices. The framework can be applied to any catchment where discharges are altered by multiple water control facilities.

### Step 1: Select a target basin

**Fig 2:**
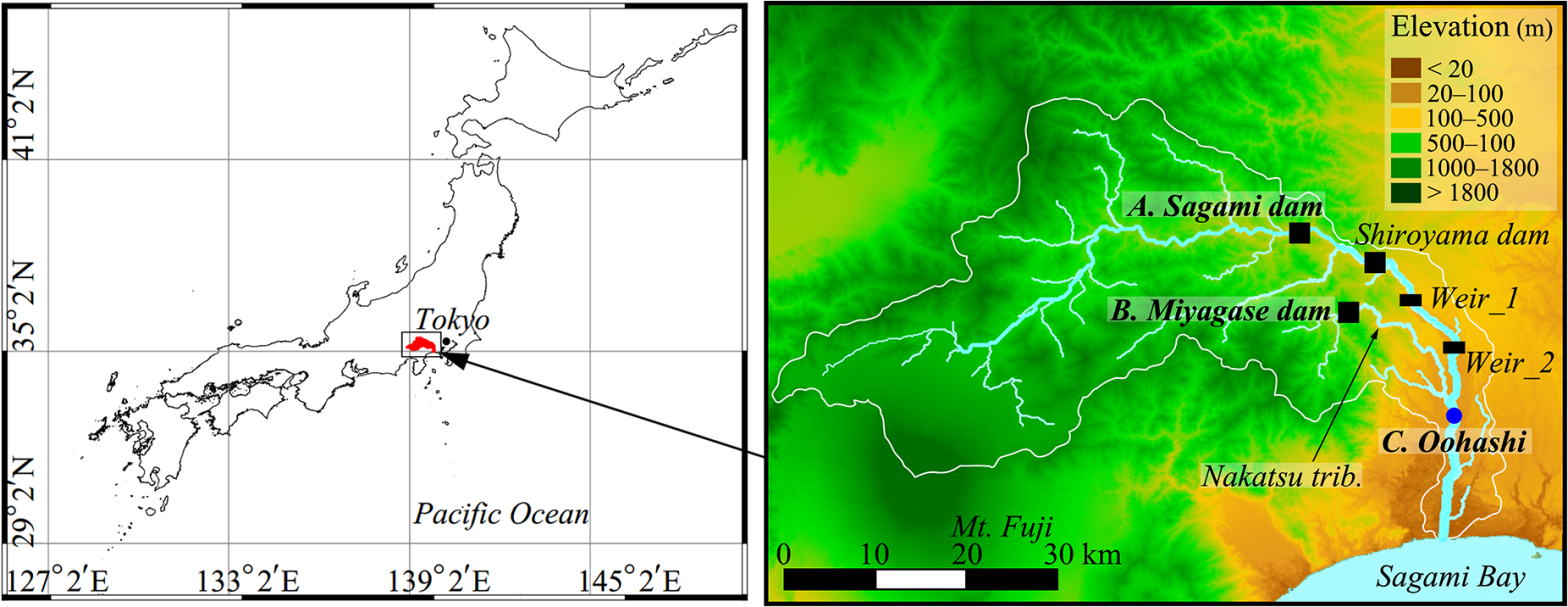
Sagami River basin in Japan. Sites for evaluating the hydrological simulation: Sagami dam (site A), Miyagase dam (site B), and Oohashi (site C).

We applied the proposed framework (Fig 1) to the Sagami River basin, which drains 1,680 km^2^ of the Kanto plain in Japan (Fig 2). The main stream of the river is 113 km long. The annual precipitation in the river basin is 1,800 mm, ranging from 1,400 mm in the north to 2,800 mm in the south. The main landuse cover comprises forest (75%) at high–middle altitudes and urban (10%) at lower altitudes. The current water allocation policy is as follows: (1) the primary water use is for hydropower generation; (2) water is supplied for domestic and industrial use for 880 million people; and (3) water is used to irrigate 95 km^2^ (9,500 ha) of agricultural land.

In this study, particular attention was paid to the downstream section in the catchment, i.e., the mainstream of Sagami River and a major tributary, Nakatsu River (elevation <500 m in Fig 2). In these areas, the discharge is modified by the Sagami dam, the Shiroyama dam, two diversion weirs on the main stream, and by the Miyagase dam on the Nakatsu tributary. Basic information regarding these water control facilities is summarized in Table 1. Given the water use, the flow conditions of the main stream above the Sagami dam and of the Nakatsu tributary above the Miyagase dam are considered natural and unaffected by human activities.

**Table 1:** Water control facilities in the Sagami River.

	Facility	Constructionyear	Usable capacity (10^6^ m^3^)	Catchment area (km^2^)	Operational purposes^a^
Mainstream	Sagami dam	1947	48.2	1129	P, W, I
	Shiroyama dam	1965	54.7	1201	F, P, W, I
	Weir 1	<1930		1336	A
	Weir 2	1940		1374	A
Tributary	Miyagase dam	2000	183	214	F, P, W, O

^a^Operational purposes. F: flood control, P: hydropower, W: water supply, I: industry, A: agriculture, O: others.

These water control facilities affect the flow regime in the following manner: Mean inflows to the Sagami and Shiroyama reservoirs are 43 m^3^ s^−1^ and 48 m^3^ s^−1^, respectively. From the reservoirs, water is withdrawn at a rate of 15 m^3^ s^−1^ throughout the year. In addition, in the downstream section of the Shiroyama dam, volumes of 5 m^3^ s^−1^ and 6 m^3^ s^−1^ are withdrawn by diversion weirs from April to September, respectively. The Miyagase dam, with mean inflow of 9 m^3^ s^−1^, regulates the seasonal flow of the tributary for flood regulation purposes. The highest monthly inflow to this dam is observed in autumn (September to November) owing to typhoons, whereas the monthly outflow is the highest in spring (March to May) to increase the capacity for flood mitigation in autumn. In this study, we evaluated the accumulated effect of the four water control facilities on the spatial variability of the flow regime.

### Step 2: Set up scenarios

The spatiotemporal variability of the daily discharge in the target basin was described under natural and altered flow conditions using a DHM. Actual flow conditions in the basin were defined as “altered” owing to the water resources management. A natural condition was defined as that wherein neither dams nor diversion weirs affect the discharge. The discharge simulation settings under both scenarios (i.e., altered and natural flow conditions) were the following: In the altered conditions, at the locations of the three dams (Fig 2), the simulated discharges were replaced by the actual release volumes from these reservoirs. The actual volumes of withdrawals at the weirs 1 and 2 (Fig 2) were subtracted from the simulated passing discharge volumes. In the natural conditions scenario, at the locations of the dams, the simulated discharges were replaced by the observed natural inflows to the reservoirs to minimize the simulation error at the target lowland region, and the withdrawals at the weirs were not considered.

### Step 3: Set up a distributed hydrological model

The DHM used in this study was a geomorphology-based hydrological model (GBHM) [23]. This model simulates the dominant hydrological processes in the basin, i.e., precipitation, canopy interception, evapotranspiration, infiltration, and percolation, as well as surface, subsurface, and groundwater level flows (Fig 1).

First, the Sagami River watershed was delineated using the gridded digital elevation data. The grid cell size was set to 250 m. All data required to run the model were aggregated and disaggregated within this cell size from their original resolution. The elevation data was the Shuttle Radar Topography Mission (SRTM) version 2, provided by the American National Geospatial-Intelligence Agency and the National Aeronautics and Space Administration (http://srtm.usgs.gov/index.php). Each grid cell includes information regarding elevation, slope, flow direction, and flow accumulation. The streamline of the Sagami River was composed of 1,335 grid cells in total.

Second, landuse and soil types were identified within the watershed to consider spatial variability of hydrological processes. Landuse type differentiates surface water retention, evapotranspiration, and horizontal–vertical movement of water [24]. We used the landuse map provided by the Earth Observation Research Center, Japan Aerospace Exploration Agency (http://www.eorc.jaxa.jp/ALOS/en/index.htm). The landuse map (version 13.08) classifies landuse cover into ten types such as evergreen forest among others (S1 Fig). This classification is based on analysis of the satellite imagery captured with the radiometer AVNIR-2. Soil type governs the dominant water movement below the land surface. Hydrological properties of soil types were assumed to vary according to geological classification. Based on the geology map provided by the Geological Survey of Japan (https://gbank.gsj.jp/seamless/zoomify_en.html), three types were identified within the watershed (sedimentary rock, volcanic rock, and accretionary complex; S1 Fig).

Third, the gridded basin was divided into 81 subbasins based on the Pfafstetter coding scheme [25], and each subbasin was subdivided into sections called flow intervals. Each flow interval contains multiple computational grid cells at the same distance band from the subbasin outlet [23]. Within flow intervals, the hillslope module calculates the lateral inflow to the stream segment. Within the stream segments, river discharge is routed using the kinematic wave equation. The DHM calculates hydrological processes at each flow interval from the uppermost subbasin and proceeds downstream.

Finally, the DHM was run by temporally-changing inputs. We used precipitation data recorded at the Japan Meteorological Agency’s 22 gauges within and around the basin (S2 Fig). The recorded hourly precipitation was spatially interpolated using splines. To calculate evapotranspiration from land surface, potential evaporation was estimated using the Priestley-Taylor’s method [26, 27]. For the estimation, we used a monthly normalized difference vegetation index, which was estimated by the Geospatial Information Authority of Japan (http://www1.gsi.go.jp/geowww/EODAS/index_e.html).

### Step 4: Calibrate DHM and evaluate the simulation accuracy

The model parameters were calibrated by comparing the simulated discharges in the altered flow conditions with the observed discharges at the three sites (Fig 2); inflows to the Sagami dam (site A) and the Miyagase dam (site B), and the discharge at the Oohashi gauging station (site C). The calibration covered the 2003–2005 period and the performance was validated for seven years (2000–2002 and 2006–2009). The three-year calibration period covered average, wet, and dry years [28]. Parameters which strongly influenced the discharge simulation were the potential evapotranspiration and Manning’s roughness coefficient for landuse cover, residual water content, saturated water content, saturated hydraulic conductivity at the topsoil layer, vertical decay factor for hydraulic conductivity [29], and Van Genuchten’s parameters [30] for soil type. Note that we firstly screened parameters influential to discharge simulation from all parameters listed in S1 Table. The rest of the parameters, which hardly influence discharge, were set to the default values.

The model performance was evaluated by calculating relative error in annual mean discharge (bias) and Nash–Sutcliffe model efficiency (NSE) [31]. Perfect matching was represented by the value of one for NSE and zero for the bias. The NSE value was calculated with the following equation:

$$NSE=1-\frac{\sum_{t=1}^{n}{\left(Q_{s}\left(t\right)-Q_{o}\left(t\right)\right)}^{2}}{\sum_{t=1}^{n}{\left(Q_{o}\left(t\right)-\bar{Q_{o}}\right)}^{2}}$$ (1)

where *t* is the computational time step, *n* is the total number of simulation time steps, *Q*(*t*) is the discharge at time *t*, subscripts *s* and *o* indicate simulated and observed discharge, respectively, and $\bar{Q_{o}}$ denotes the observed mean discharge. NSE is one of the most popular indices for evaluating models. However, it is sensitive to high extreme values [32]. Therefore, NSE values based on the log_10_-transformed discharge (NSE_log) and the inverse of the discharge (NSE_inverse) were also evaluated. The log-transformed discharge was considered to represent the variability balanced at middle and high pulses [22, 33]. The NSE_inverse index is used to evaluate the accuracy of extremely low flow [34].

The accuracy of the flow aspects is strongly influenced by the calibration strategy of a model [22]. In this study, calibration strategy particularly aimed to simulate floods, high flows, and monthly flows, which relate the primary problems in this river. In addition, we attempted to pay attention to low flows, minimum flow, and long-term bias. The shuffle complex evolution method developed at the University of Arizona (SCE-UA) [35] was used in the calibration. The DHM was calibrated three times using different criteria. First, NSE was maximized after minimizing the bias. Second, constraining the bias at <10% and NSE at >0.8, we maximized NSE_log. Third, we maximized NSE_inverse, while constraining the NSE_log at >0.6. In this manner, we simulated the various aspects of flow characteristics reasonably well.

### Step 5: Transform the simulated daily river discharge to a set of ecologically-relevant hydrologic indices and evaluate them

The Indicators of Hydrologic Alteration (IHA) software package [36, 37] was used to evaluate 33 flow characteristics in five ecologically relevant categories: 1) the magnitude of monthly median flows; 2) the magnitude and duration of annual extremes, i.e., maximum and minimum flows; 3) the timing of the annual extremes; 4) the frequency and duration of high and low pulses; and 5) the rate of change. Thirty-three hydrologic indices were calculated from the observed and simulated discharges for each year from 2000 to 2009. We did not evaluate the number of zero-flow days owing to perennial flow, i.e., there was no zero flow. The thresholds for low and high flow pulses (category 4) were defined as the 25th and 75th percentiles, respectively, of the 20-year (1990–2009) daily natural flows simulated by the DHM [38]. The calculation of these indices was performed using R 3.1.0 [39] combined with the IHA package (ver. 0.2–41; https://r-forge.r-project.org/projects/iha/).

The flow indices derived from the observations and simulations were compared by applying the two-sample Kolmogorov–Smirnov (K–S) test and Pearson’s correlation analysis for individual flow indices. A similar method was adopted in [22]. In this context, the K–S test evaluated the null hypothesis that samples from the observations and simulations were collected from the same distribution for each flow index. Thus, for example, a K–S test result showing statistical insignificance (α = 0.05) suggests that the corresponding flow index is reasonably simulated by the DHM without significant bias.

Based on the two evaluations, we grouped the IHA indices in 1) good indices, with the K–S test showing statistically insignificant differences between observations and simulations and a correlation coefficient of >0.6 at the three sites, A–C; 2) moderate indices, with the K–S test showing statistically insignificance and a correlation coefficient of >0.4 at two or three sites; and 3) poor indices, i.e., the remaining indices. Note that the criteria used in this study can be adjusted according to purpose of use. The above categorization represents the reliability of the spatial variability of the IHA indices, which is estimated in the following section.

### Step 6: Visualize and evaluate the spatial patterns of altered flow regimes comparing them with the natural flow condition

To evaluate the spatial patterns of flow regimes and the alterations of the stream network, the 33 IHA indices were also calculated every year based on the 10-year simulated discharges at all computational grids (1,335 grid cells at 250 m resolution) under natural and altered conditions (Fig 1). Threshold values for low and high flows (i.e., 25^th^ and 75^th^ percentiles of discharge) were derived from the 20-year simulated natural flow at every grid cell. To compare the general patterns under both conditions, the median of the 10-year index values at every grid cell was calculated for all IHA indices. Then, the alteration of flow regimes was evaluated at the downstream sections of the two dams (i.e., sites A and B in Fig 2), considering the accuracy of each hydrological index. The degree of alteration for each flow index was calculated as the relative change in the median values. The calculated indices and the degree of alteration were assigned to the stream network using the QGIS version 2.2.0 for visualization [40].

## Results and Discussion

### Calibration and validation of the DHM

**Fig 3:**
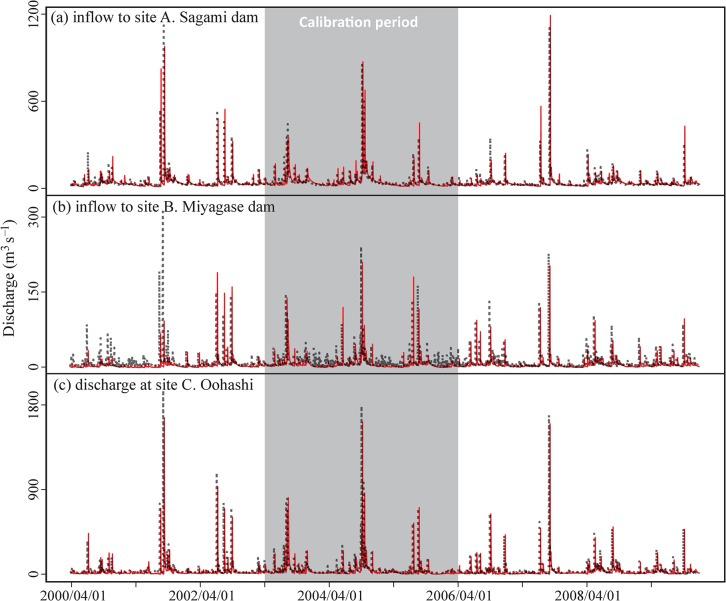
Observed (black) and simulated (red) discharges at the three gauged sites. The calibration period is shown in gray and the validation period is shown in white.

The calibration and validation results show that the DHM slightly underestimates the total water volume, with a bias ranging from −4% to −9% (Table 2). The mean and the range of the observed and simulated discharge increase in proportion to the catchment area (i.e., site B < A < C), whereas the high flow patterns were similar among the sites (Fig 3). High flows were better simulated than low flows as NSE decreased in the order of NSE > NSE_log > NSE_inverse. Based on the performance indices (bias, NSE, and NSE_log), the discharges at site C were most accurately simulated, followed by site A and then site B (e.g., NSE were 0.97, 0.90, and 0.69, respectively).

**Table 2:** Model calibration results at the three evaluation sites. The calibration period was 2003–2005, and the validation period was the remaining target years from 2000 to 2009.

Statistical indices	Performance evaluation: calibration (validation)					
	A. Sagami reservoir		B. Miyagase reservoir		C. Oohashi	
bias	−0.04	(−0.07)	−0.06	(−0.09)	−0.05	(−0.06)
NSE^a^	0.93	(0.90)	0.85	(0.69)	0.98	(0.97)
NSE_log^b^	0.78	(0.66)	0.63	(0.63)	0.89	(0.78)
NSE_inverse^c^	0.33	(0.01)	0.44	(0.29)	0.58	(0.25)

^a^Nash–Sutcliffe model efficiency

^b^NSE calculated for log_10_-transformed discharge

^c^NSE calculated for inverse-transformed discharge

### Categorization of simulated flow characteristics based on accuracy

**Fig 4:**
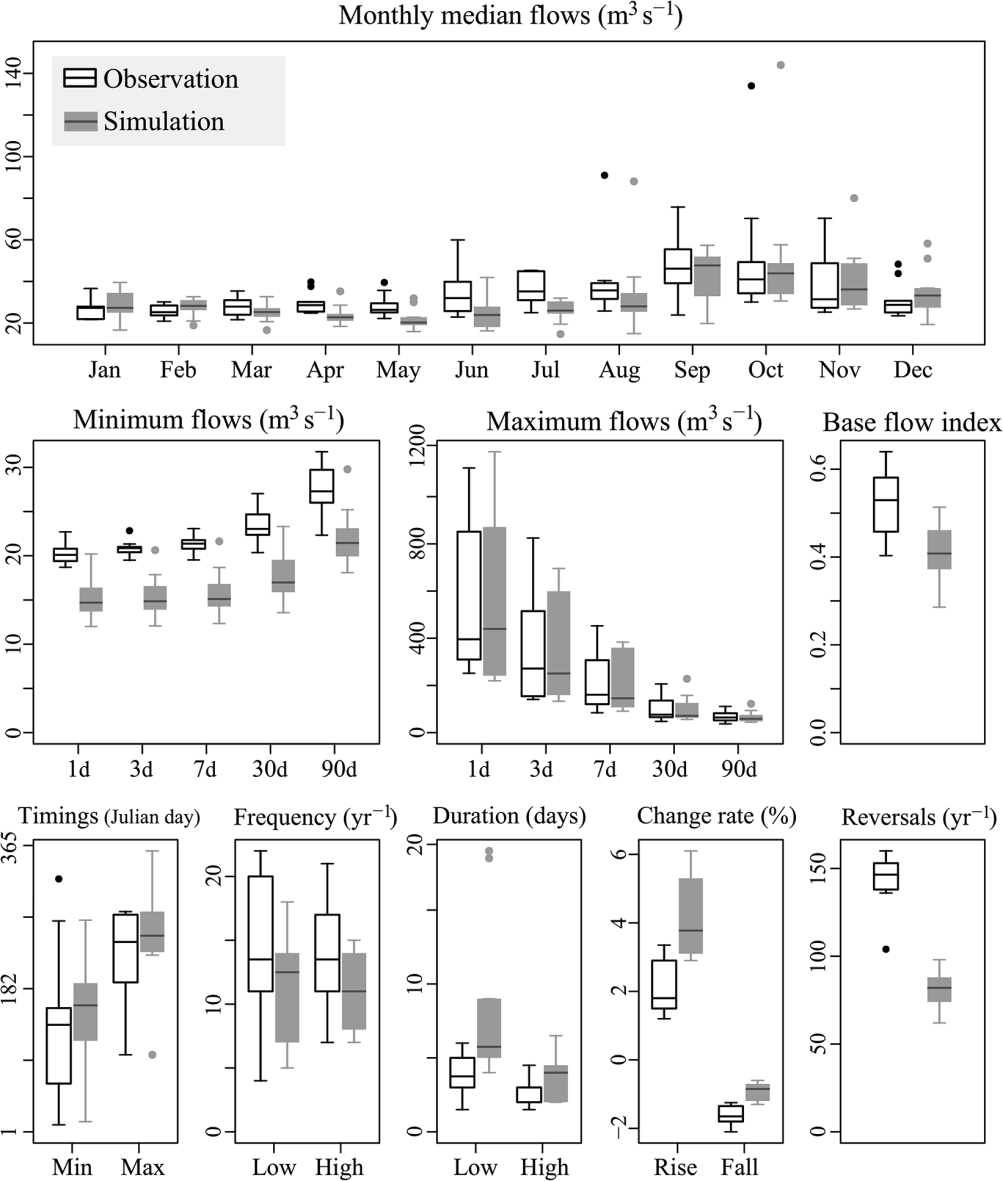
Box-and-whisker plots for hydrologic indices at Sagami dam. Indices were calculated from observed (black) and simulated (gray) inflow to the Sagami dam (site A). The lines at the bottom, middle, and top of the boxes represent the 25th, 50th, and 75th percentile, respectively. Vertical bars represent 10th and 90th percentiles.

**Fig 5:**
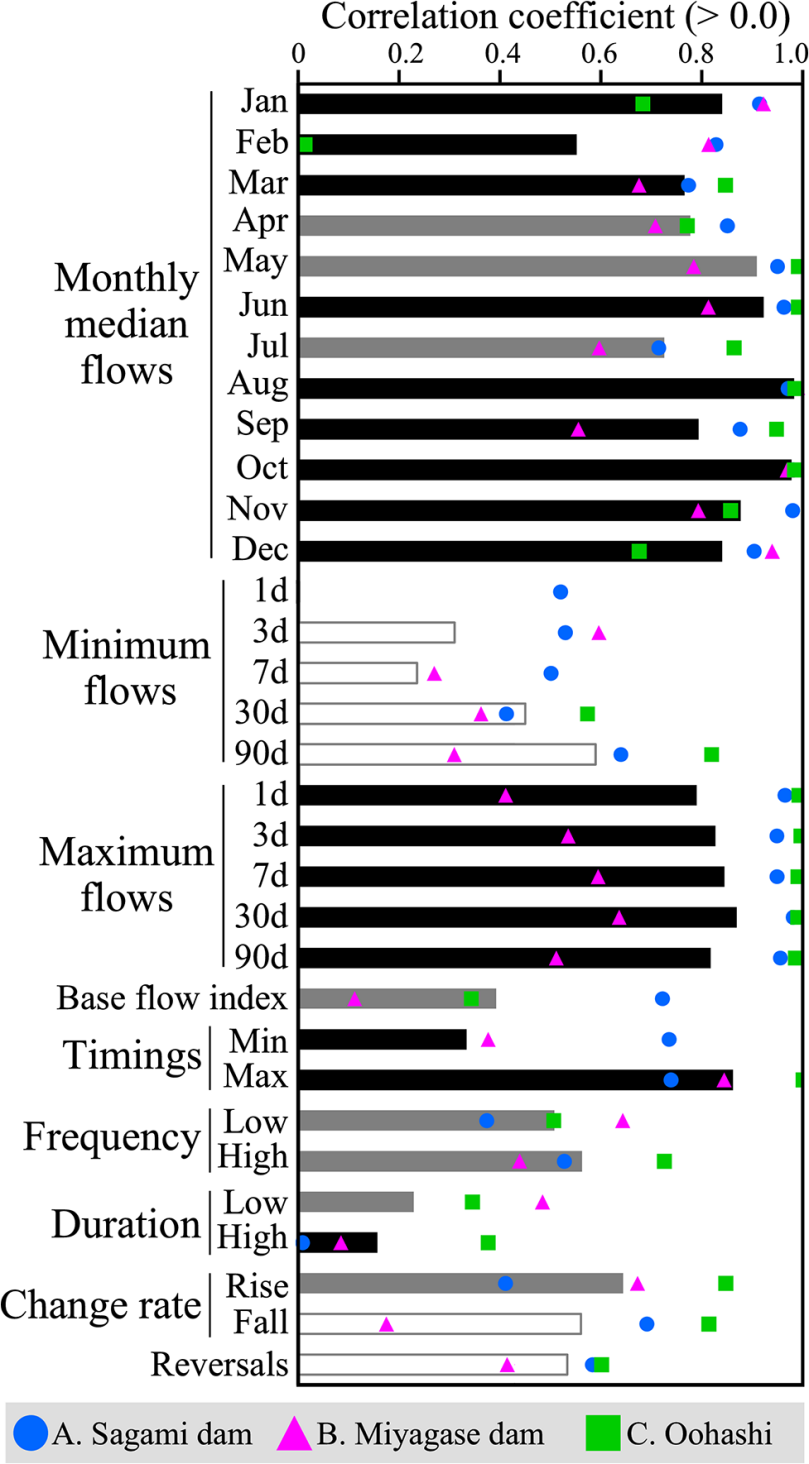
Flow indices calculated from observed and simulated discharges at the three sites. Bars indicate mean correlation coefficients across the three sites (only positive values are shown). The black, gray, and white bars correspond to the number of non-significant results from the Kolmogorov–Smirnov test (3, 2, and 1/0 respectively).

The IHA hydrologic indices calculated from the observed and simulated discharges were compared at the three sites (Fig 4 for site A; S3 Fig for the other sites). The indices were then classified (Table 3) based on the results of the K–S test and correlation analysis (Fig 5). Monthly median flows and maximum flows with their timing, along with the frequencies of high and low flows, were categorized as either “good” or “moderate.” In contrast, minimum flows with their timing, durations of high and low flows, and rate of change were ranked as “moderate” or “poor.” It should be stressed that the use of model-estimated flow indices categorized as “poor” may mislead implications or give wrong predictions [18] and thus special attention is needed.

**Table 3:** Categorization of hydrologic indices based on the results of the Kolmogorov–Smirnov test and Pearson’s correlation analysis. See the method for the definition of the three categories (good, moderate, and poor).

Rank	Hydrologic indices
Good	Monthly median flows in January, March, June, August, October, November, and December; 30d maximum flow; timing of 1d maximum flow
Moderate	Monthly median flows in February, April, May, July, and September; 1d, 3d, 7d, and 90d maximum flows; 30d and 90d minimum flows; frequency of low and high pulses; rise rate
Poor	1 d, 3 d, and 7 d minimum flows; timing of 1 d minimum flow; base flow index; duration of low and high pulses; fall rate; the number of reversals

Our results are consistent with those in [22], showing better performance in reproducing the flow indices related to high flows than low-flow related indices. This is due to the prioritization of the calibration for high flows, i.e., NSE. In contrast, rise and fall rates, reversals, durations of flow pulses were not simulated well in both studies. Differences in the simulation accuracy were found in the frequency of high pulses and minimum flows. High flow pulses in the target basin are mainly caused by strong rainfall events, and such hydrological processes are usually well simulated by a DHM. On the other hand, high flow pulses driven by snowmelt require the simulation of the energy flux which is more complex and difficult to model. The poor simulation of minimum flows is attributed to observation error, limitations of groundwater modeling, and our calibration strategy with the priority as NSE > NSE_log > NSE_inverse [22].

The K–S test and correlation analysis evaluated different aspects of the simulation accuracy. For instance, the K–S test showed statistical significance for monthly median flows in April, May, and July at site A, suggesting poor simulation accuracy for these flows. Indeed, these monthly flows were clearly underestimated (the uppermost panel in Fig 4). In contrast, the simulated and observed values for these indices were highly correlated (r > 0.7 in Fig 5). The results suggest that the simulation was able to capture the interannual variabilities in these monthly flows despite underestimating the absolute discharge volumes.

Differences in the simulation accuracy among indices were partly attributed to the calibration strategy for simulating the discharges, i.e., NSE > NSE_log > NSE_inverse. The selection of the performance criteria for calibrating a hydrological model should be made in accordance with the purpose and objectives of the simulation (see several evaluation indicators in [33]). For example, a DHM can be calibrated by including specific flow indices, such as the monthly median flows, as performance criteria. In addition, to improve the overall accuracy of the discharge simulation, including high and low flows, the calibration of water movement and storage under the surface, i.e., soil moisture and ground water table, should be considered [41, 42].

### Evaluation of spatial patterns in altered flow regimes

**Fig 6:**
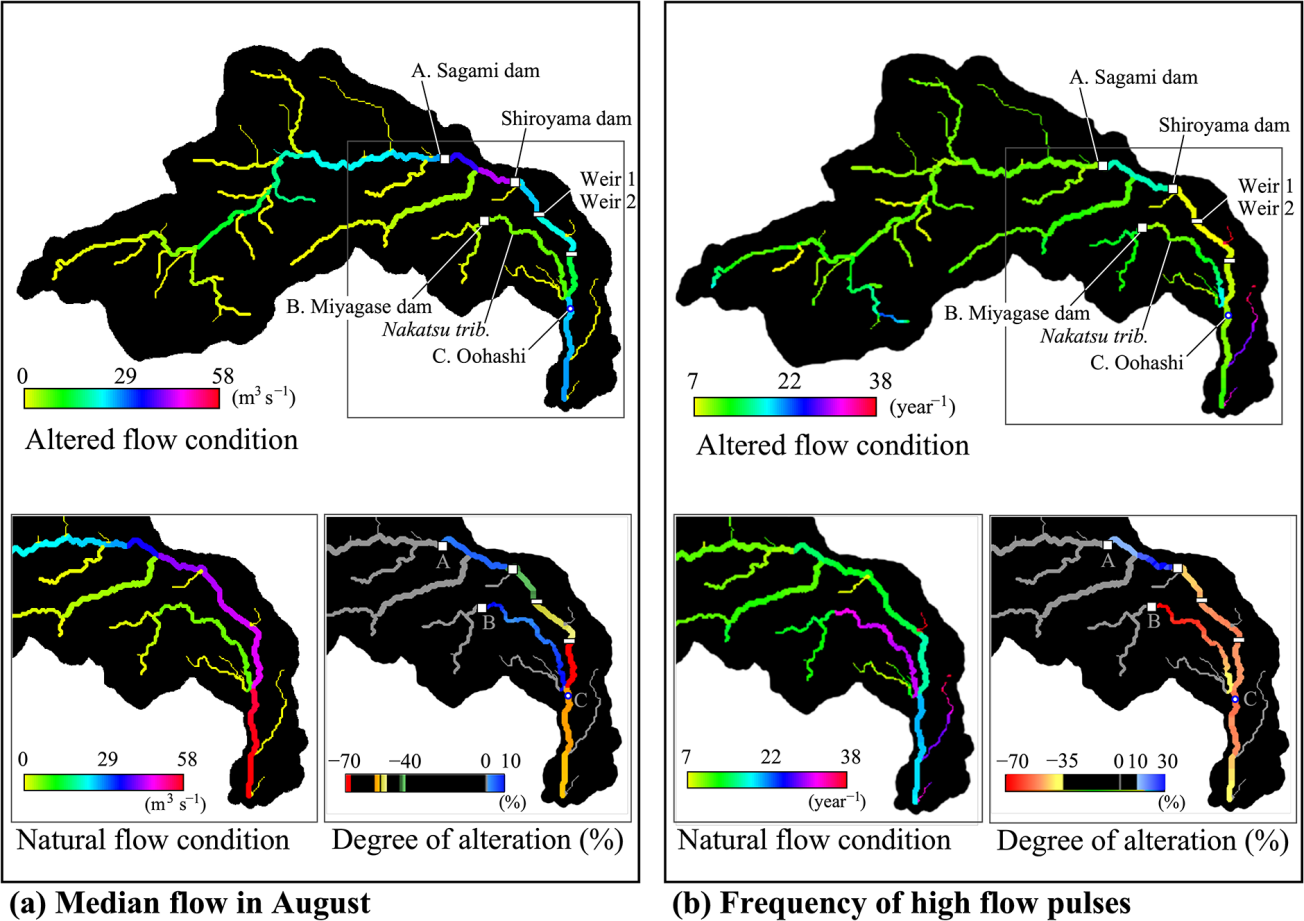
Spatial patterns of the flow regime. (a) median flow in August and (b) frequency of high flow pulses in the altered (upper) and natural (lower-left) conditions. Lower-right panels show the degrees of alteration (relative changes from the natural flow condition to the altered one).

As examples, the spatial patterns of two flow characteristics as well as their degree of alterations (monthly median flow in August and frequency of high pulses) were presented and discussed below (Fig 6; S4 Fig for the other 30 indices). Note that the reliability of the individual maps including the figures in S4 should be evaluated based on the simulation accuracy described above. Based on the performance evaluation results, the August median flow and frequency of high pulses were evaluated as “good” and “moderate,” respectively. The median flow in August represented the summer flow conditions when water demand increased because of irrigation and domestic use. On the other hand, the reduction in the frequency of the high flow pulses is a major environmental problem in the Nakatsu tributary that is responsible for the stabilization of sandbars, periphyton growth, and silt deposition on the riverbed since the start of the operation of the Miyagase dam [43].

The median flow in August monotonically increased as the catchment size increased from the upper region along the main stream toward site A (28 m^3^ s^−1^, Fig 6A). The highest volumes were observed in the section between the Sagami dam (site A) and the Shiroyama dam (ranging from 36.5 to 43.5 m^3^ s^−1^). This was attributed to the inflow from the large tributary and the slight increase in the release from the Sagami dam compared with the natural flow conditions. River water was withdrawn at the Shiroyama dam and the two diversion weirs located downstream. The maximum reduction (−70%) was observed at the section between weir_2 and the river’s confluence with the Nakatsu tributary. As a result of a 10% increase in inflows from the tributary caused by the Miyagase dam at site B, the ratio of reduction at its confluence with the mainstream was mitigated from 70% to 54%. This indicates that the alteration of flow regimes in tributaries affects the flow regime in the mainstream.

The frequency of high flow pulses, which is expected to change as a result of dam operations for flood control (i.e., reduction of the peak of high flows), was spatially homogeneous compared with the August median flow (Fig 6B). The mean and standard deviation of the frequency on the stream network was 12 and 5.3 times per year, respectively. Remarkably high values (>30) were found at the two small tributaries in the Eastern part of the catchment. This is due to the combination of calibrated soil and landuse properties but had almost no effect on the mainstream. The frequency of high flows decreased most at the Miyagase dam (−70%). The mainstream below the Shiroyama dam experienced a moderate decrease in the frequency of high flows (between −46% and −55%). The highest decrease at the Miyagase dam likely stems from the large capacity of its reservoir (183 × 10^6^ m^3^), which is three times larger than that of the Sagami (48 × 10^6^ m^3^) and Shiroyama reservoirs (55 × 10^6^ m^3^). The reduction ratios gradually decreased downstream with distance from the Miyagase dam toward the confluence with the mainstream (from −70% to −36%, Fig 6B), supporting the serial discontinuity concept [44].

Comparing the degrees of alterations in the two indices uncovered that their spatial patterns of alteration differ. The strongest decrease in the August median flow (−70%) was detected in the mainstream, whereas the strongest reduction in the frequency of high pulses (−70%) occurred in the Nakatsu tributary (red pattern in Fig 6A and 6B). This likely resulted from the combination of the various purposes of the water resources managements in the basin (Table 1). These results indicate that flow regimes at a single station (e.g., the river outlet) are not always representative of a river network, and thus, the evaluation of the spatial pattern is necessary to capture the changes in the flow regime of a basin.

The framework (Fig 1) can be used flexibly to evaluate the spatial pattern of flow regimes. For example, the spatial pattern of the degree of alteration can be quantitatively evaluated by correlation analysis (S1 File). Such analyses enable more detailed evaluation of the effects of water control facilities on the flow regime of mainstream as well as the effect of tributaries’ inflows. In addition, various scenario analyses can be conducted. For example, we can simulate and compare various reservoir operational rules, which affect flow regimes in different manners, to explore a balance between ecosystem and human needs [45].

## Conclusions

Spatial patterns of altered flow regimes along the Sagami River were quantitatively evaluated using the DHM. The simulation accuracy of the hydrologic indices was generally consistent with a previous study [22] that demonstrated better performance for high-flow indices than low-flow indices owing to the DHM calibration priority for high flows. The results reveal that evaluating the spatial patterns of flow regimes readily identifies locations where flow aspects are strongly altered by the multiple water control facilities distributed on a river network. The strongest alterations in different aspects of flow regimes did not occur at the same location, implying that evaluation only at discharge gauges is insufficient to capture alteration in the flow characteristics of a stream network. Thus, the spatial patterns of flow regimes on a river network should be understood when evaluating the alterations caused by multiple water control facilities.

## Supporting Information

S1 Fig Landuse and soil map. (PDF)

S2 Fig The location of precipitation measurement. (PDF)

S3 Fig Box-and-whisker plots for hydrologic indices at sites B and C. (PDF)

S4 Fig Spatial patterns of all the IHA indices. (PDF)

S1 File Spatial correlation analysis for Fig 6. (PDF)

S1 Table Parameters for landuse and soil types. (PDF)
