# Supplementary figures and images for: Evaluation of Spatial Pattern of Altered Flow Regimes on a River Network Using a Distributed Hydrological Model
Source: PLoS One. 2015 Jul 24;10(7):e0133833. doi: 10.1371/journal.pone.0133833 (PMC4514816; doi:10.1371/journal.pone.0133833)

(a) Landuse map

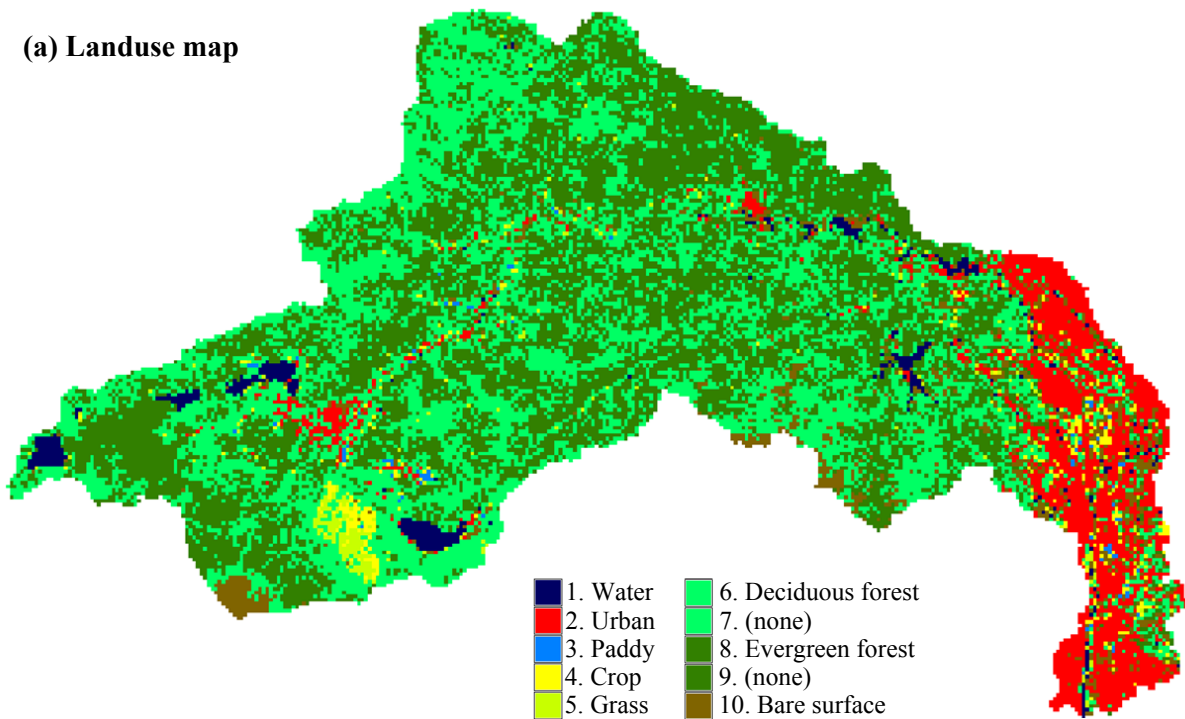

(b) Soil map

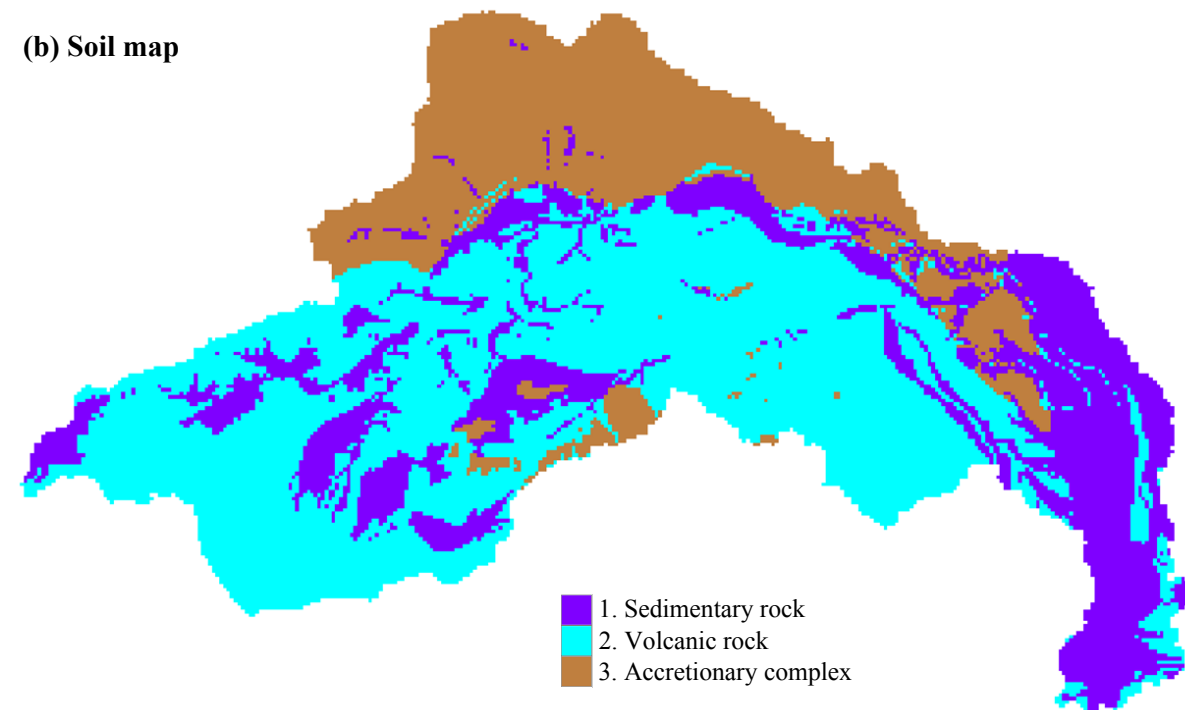

Supplement: S1 Fig — (PDF) [file pone.0133833.s001.pdf]

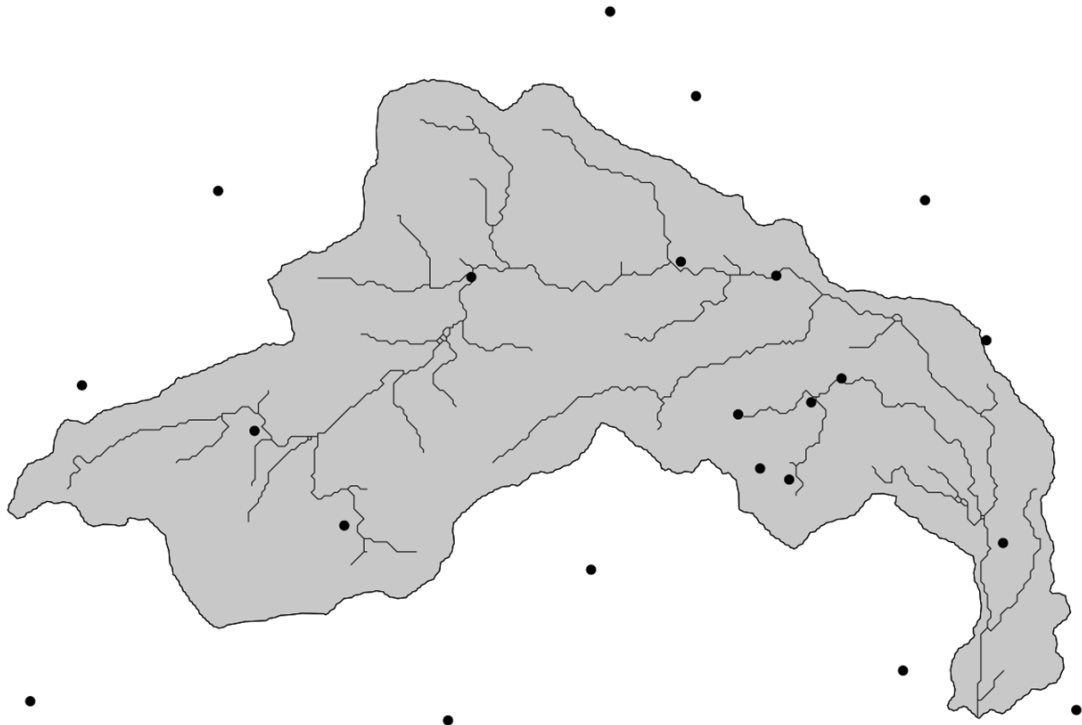

Supplement: S2 Fig — (PDF) [file pone.0133833.s002.pdf]
