# Supplementary material for: Evaluation of Spatial Pattern of Altered Flow Regimes on a River Network Using a Distributed Hydrological Model
Source: PLoS One. 2015 Jul 24;10(7):e0133833. doi: 10.1371/journal.pone.0133833 (PMC4514816; doi:10.1371/journal.pone.0133833)
Supplement: S3 Fig — (PDF) [file pone.0133833.s003.pdf]

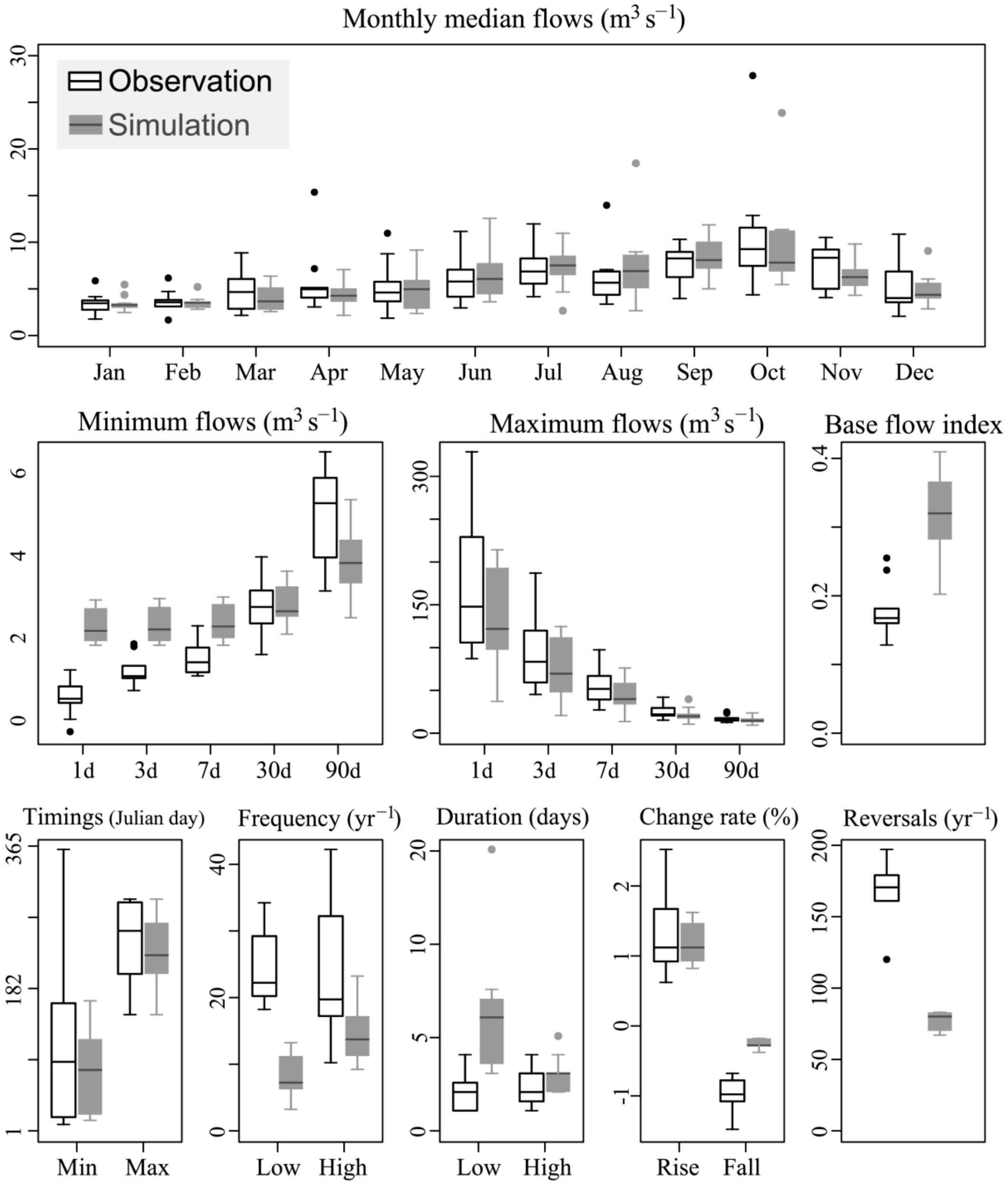

(a) Box-whisker plots for flow indices calculated from observed (black) and simulated (gray) inflow to Miyagase dam (site B). The lines at the bottom, middle, and top of the boxes represent the 25th, 50th, and 75th percentiles of the values, respectively. Vertical bars represent 10th and 90th percentiles.

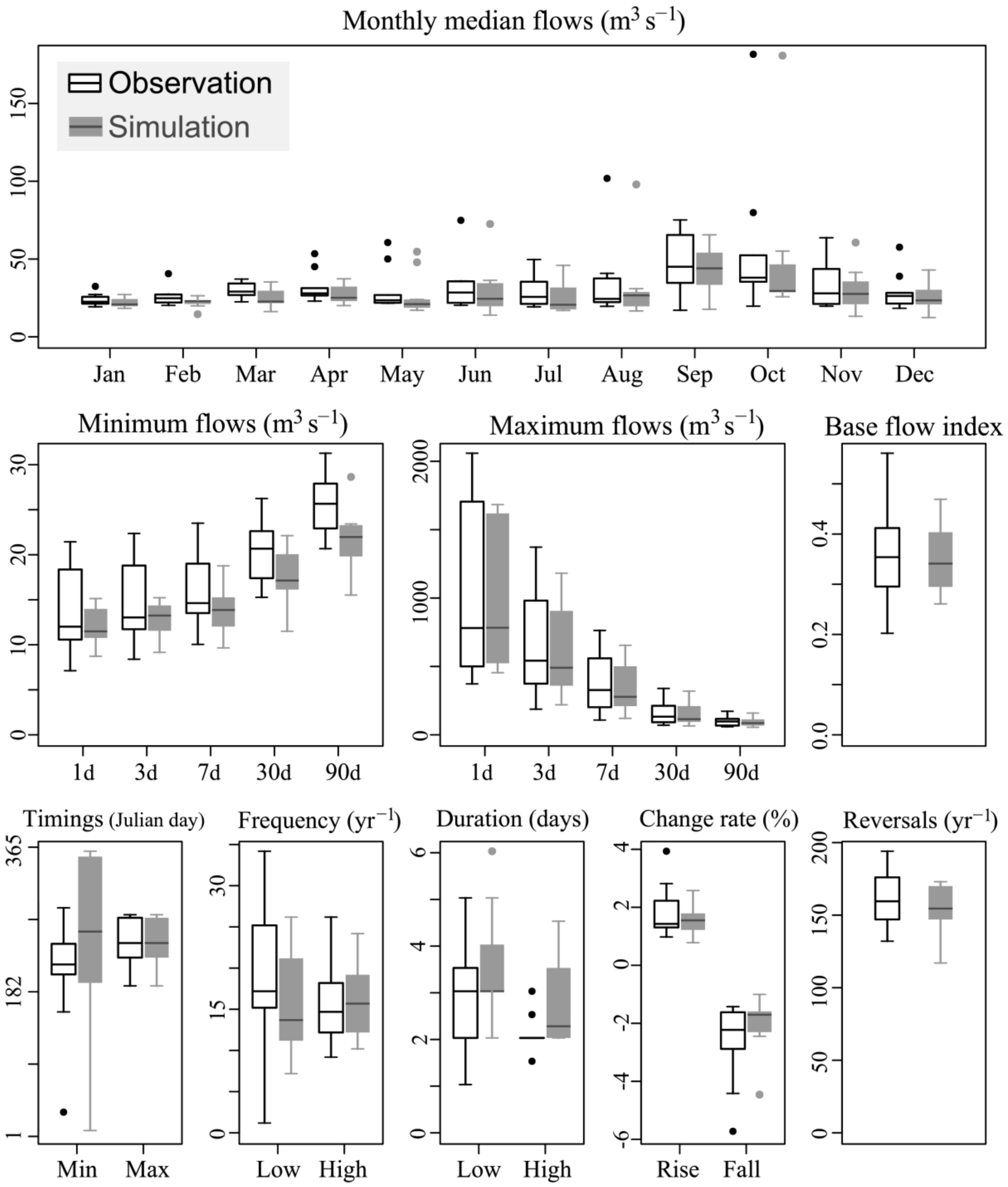

(b) Box-whisker plots for flow indices calculated from observed (black) and simulated (gray) discharge at Oohashi gauging station (site C). The lines at the bottom, middle, and top of the boxes represent the 25th, 50th, and 75th percentiles of the values, respectively. Vertical bars represent 10th and 90th percentiles.
