# Supplementary material for: Evaluation of Spatial Pattern of Altered Flow Regimes on a River Network Using a Distributed Hydrological Model
Source: PLoS One. 2015 Jul 24;10(7):e0133833. doi: 10.1371/journal.pone.0133833 (PMC4514816; doi:10.1371/journal.pone.0133833)

List of IHA indices

(1) January median flow (m^3 s^{-1})
(2) February median flow (m^3 s^{-1})
(3) March median flow (m^3 s^{-1})
(4) April median flow (m^3 s^{-1})
(5) May median flow (m^3 s^{-1})
(6) June median flow (m^3 s^{-1})
(7) July median flow (m^3 s^{-1})
(8) August median flow (m^3 s^{-1})
(9) September median flow (m^3 s^{-1})
(10) October median flow (m^3 s^{-1})
(11) November median flow (m^3 s^{-1})
(12) December median flow (m^3 s^{-1})

(13) 1 day minimum flow (m^3 s^{-1})
(14) 3 day minimum flow (m^3 s^{-1})
(15) 7 day minimum flow (m^3 s^{-1})
(16) 30 day minimum flow (m^3 s^{-1})
(17) 90 day minimum flow (m^3 s^{-1})

(18) 1 day maximum flow (m^3 s^{-1})
(19) 3 day maximum flow (m^3 s^{-1})
(20) 7 day maximum flow (m^3 s^{-1})
(21) 30 day maximum flow (m^3 s^{-1})
(22) 90 day maximum flow (m^3 s^{-1})

(23) Base flow index

(24) Timing of minimum flow (Julian day)
(25) Timing of maximum flow (Julian day)

(26) Frequency of high pulse (times $year^{-1}$)
(27) Frequency of low pulse (times $year^{-1}$)

(28) Duration of high pulse (days)
(29) Duration of low pulse (days)

(30) Rise rate (%)
(31) Fall rate (%)
(32) Reversals (times)

Note that number of zero flow days were not calculated because of perennial flow.

Natural flow scenario (Left) and altered flow scenario (Right)

(1) January median flow ($m^3\ s^{-1}$)

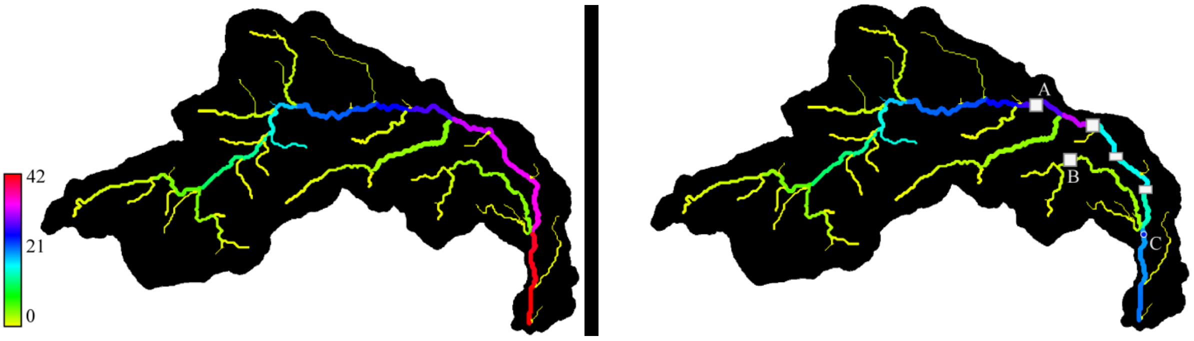

(2) February median flow ($m^3\ s^{-1}$)

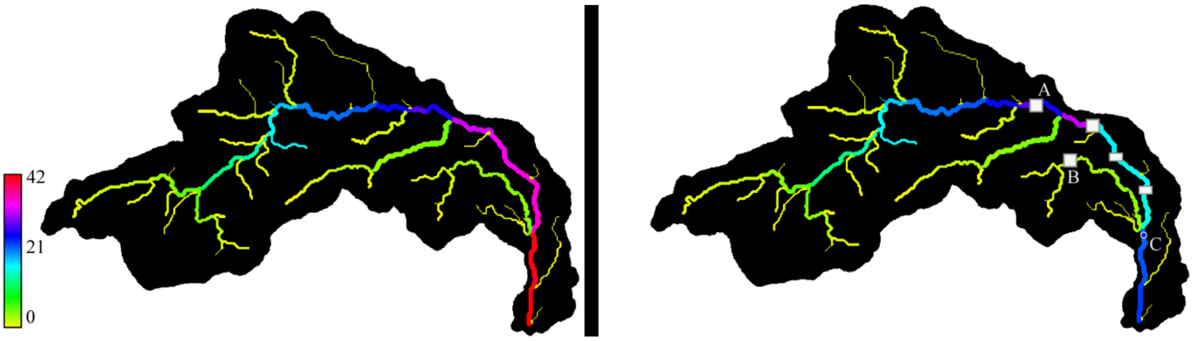

(3) March median flow ($m^3\ s^{-1}$)

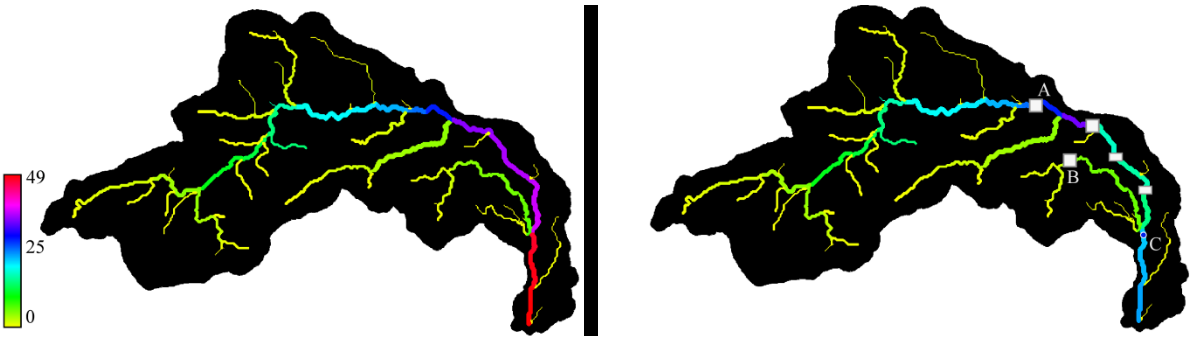

(4) April median flow ($m^3\ s^{-1}$)

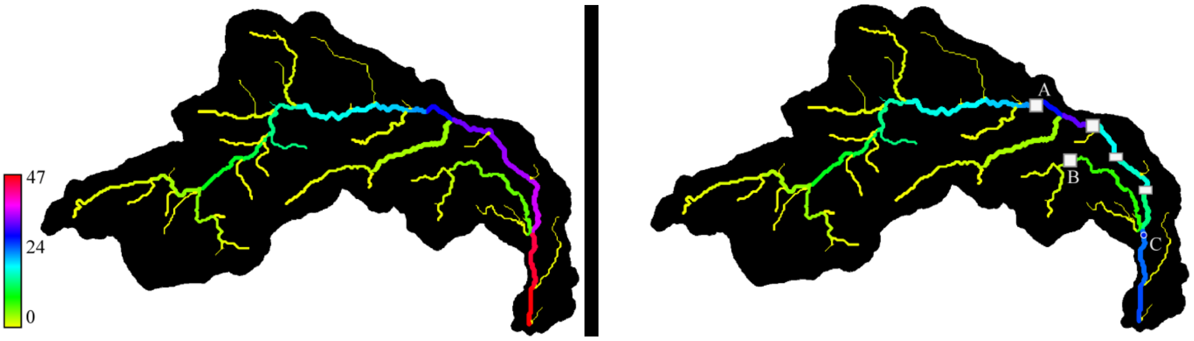

(5) May median flow ($m^3\ s^{-1}$)

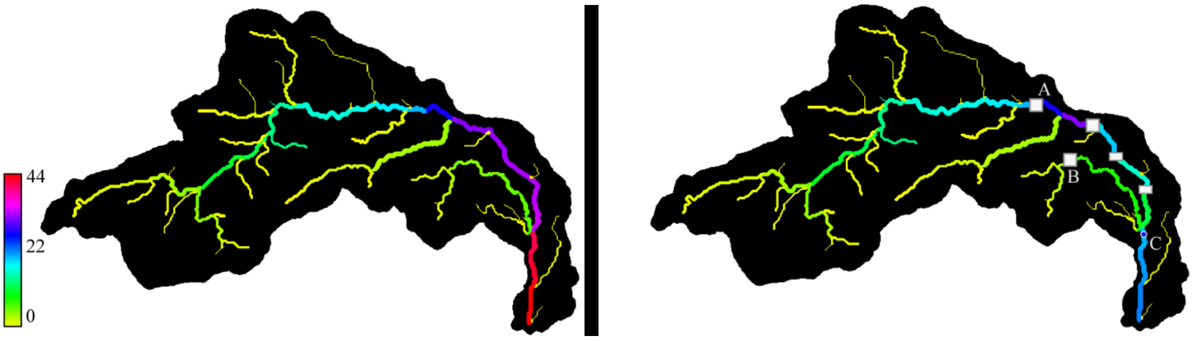

(6) June median flow ($m^3\ s^{-1}$)

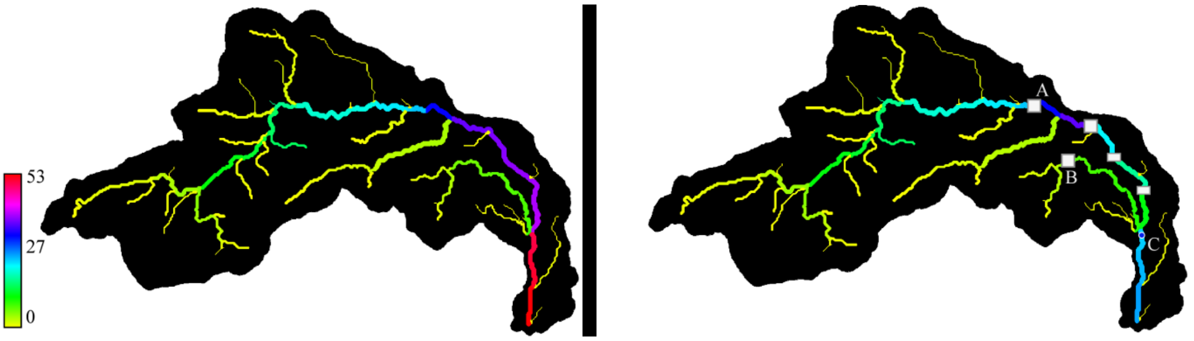

(7) July median flow ($m^3\ s^{-1}$)

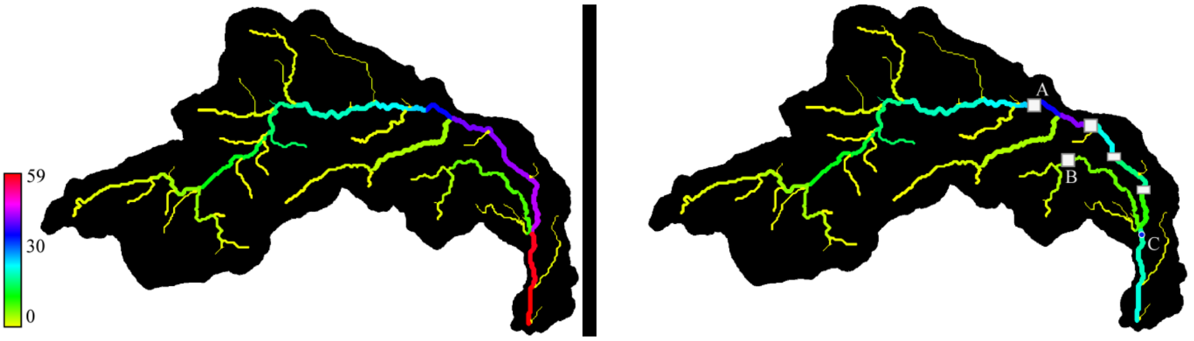

(8) August median flow ($m^3\ s^{-1}$)

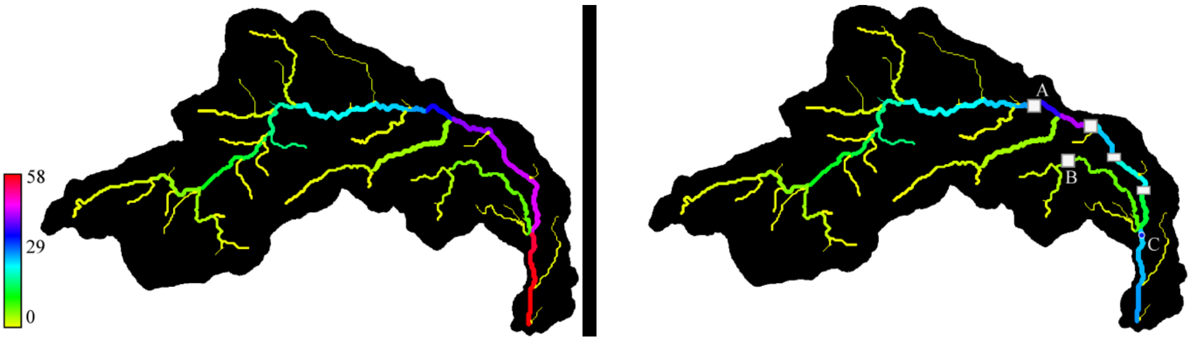

(9) September median flow ($m^3\ s^{-1}$)

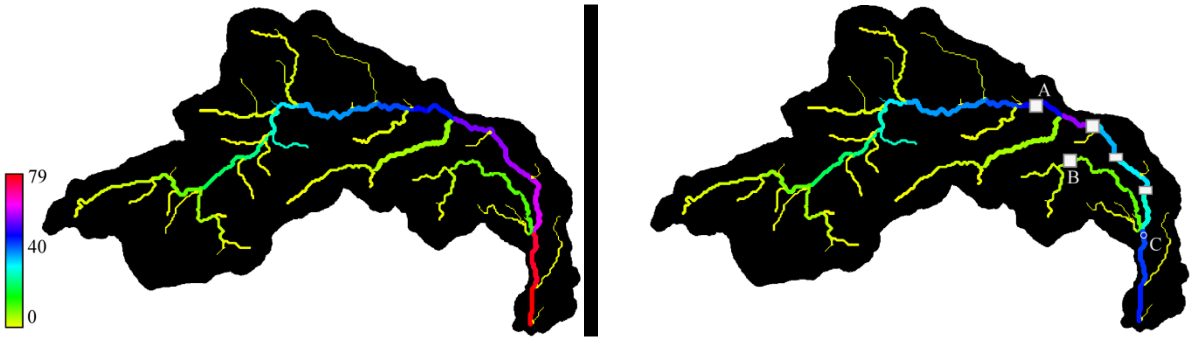

(10) October median flow ($m^3\ s^{-1}$)

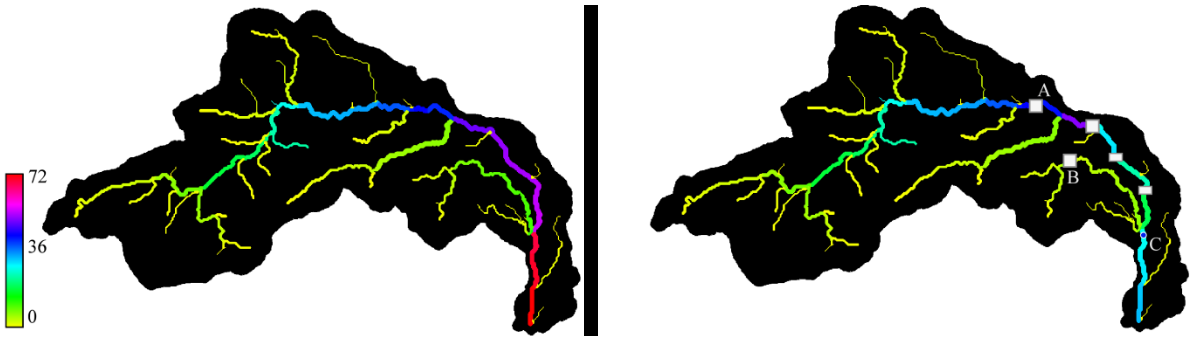

(11) November median flow ($m^3\ s^{-1}$)

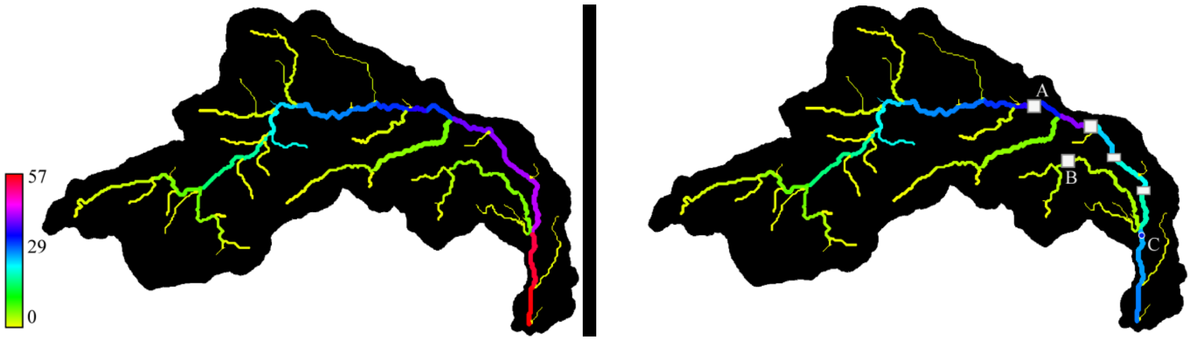

(12) December median flow ($m^3\ s^{-1}$)

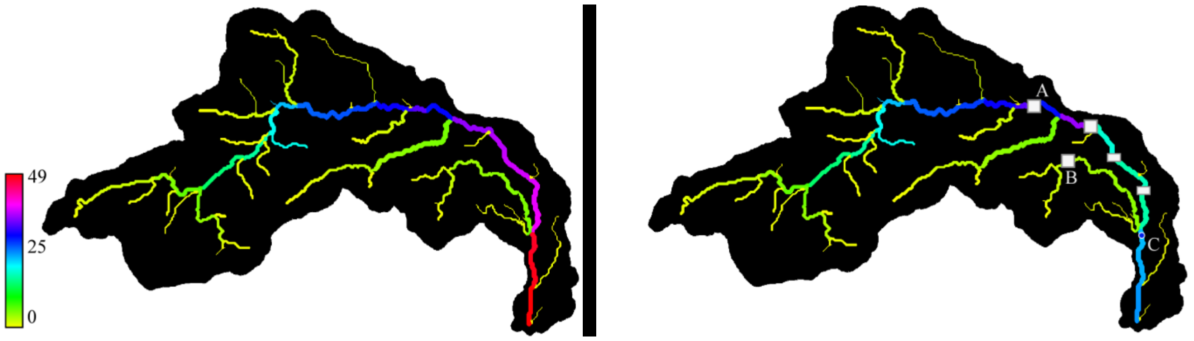

(13) 1 day minimum flow ($m^3\ s^{-1}$)

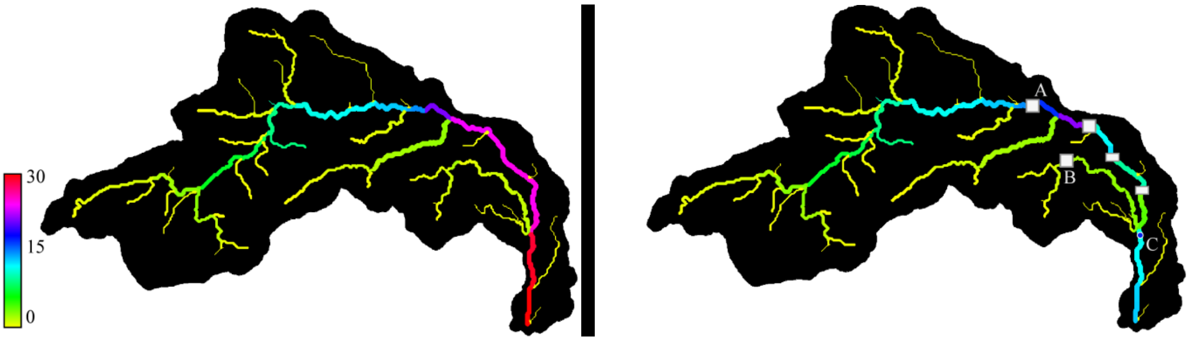

(14) 3 day minimum flow ($m^3\ s^{-1}$)

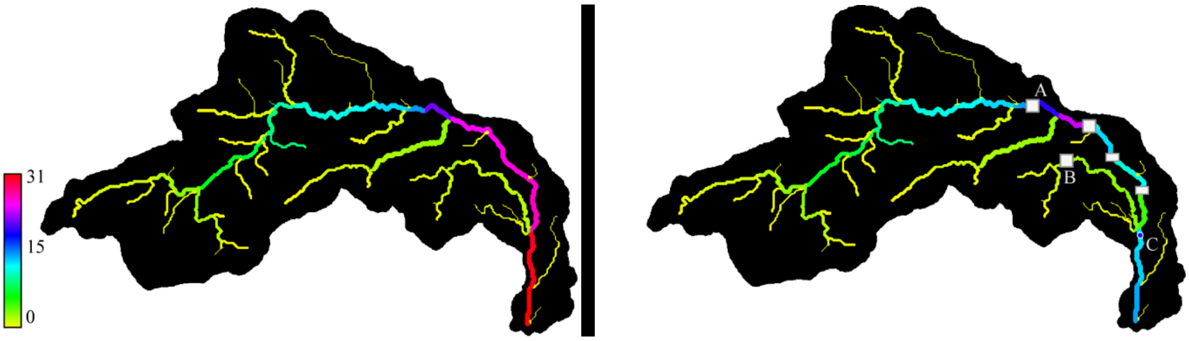

(15) 7 day minimum flow ($m^3\ s^{-1}$)

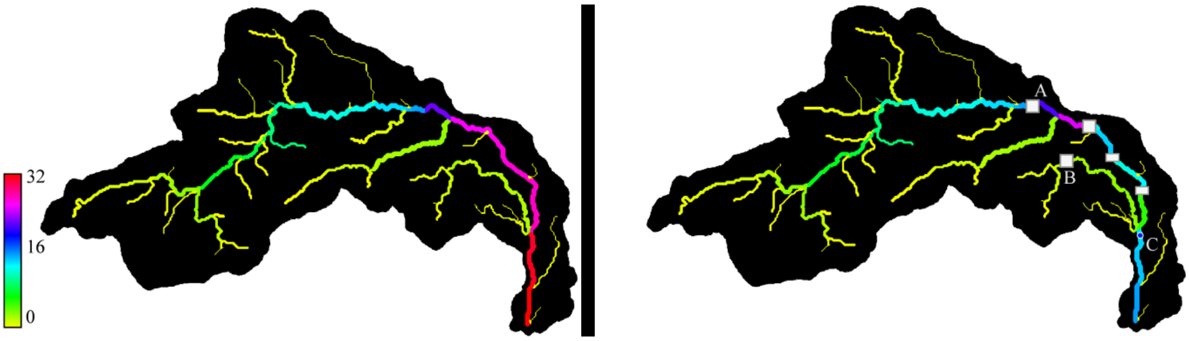

(16) 30 day minimum flow ($m^3\ s^{-1}$)

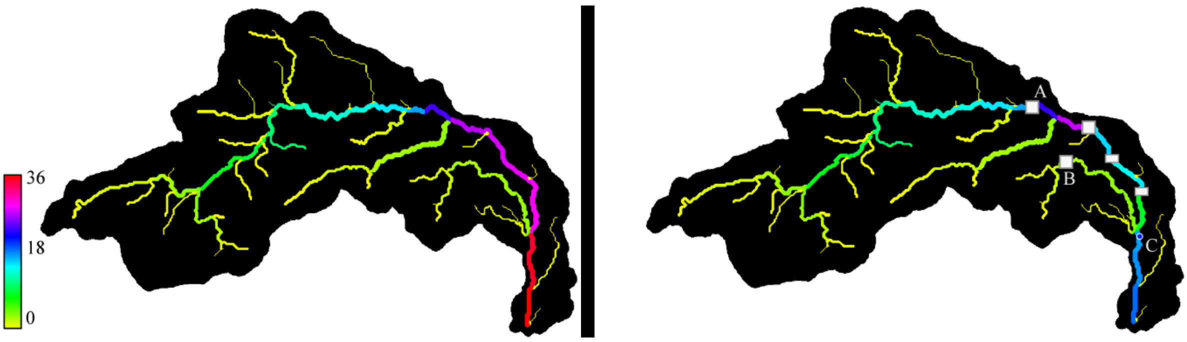

(17) 90 day minimum flow (m^3 s^{-1})

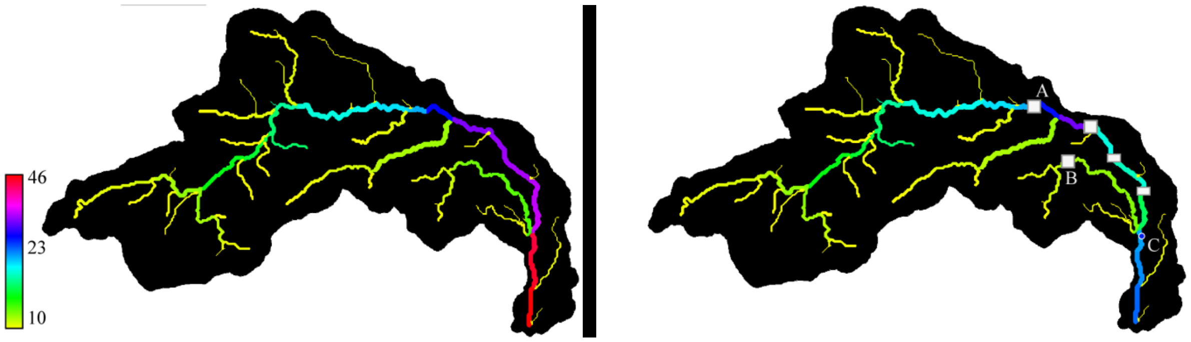

(18) 1 day maximum flow (m^3 s^{-1})

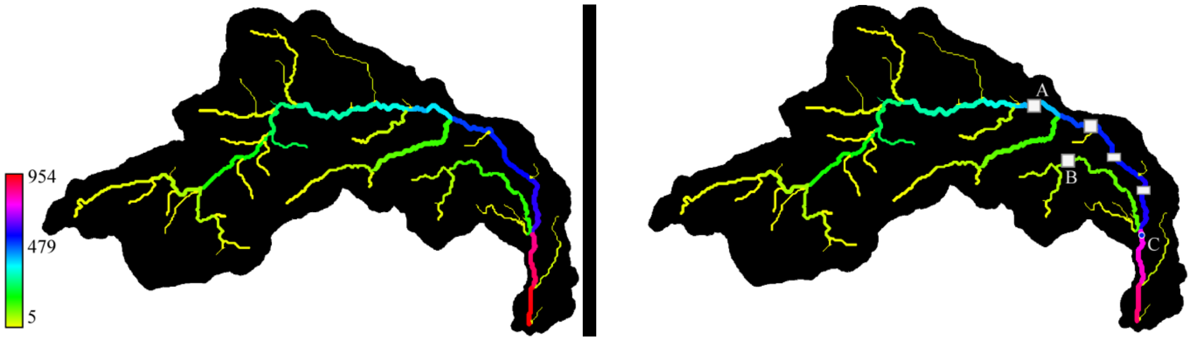

(19) 3 day maximum flow (m^3 s^{-1})

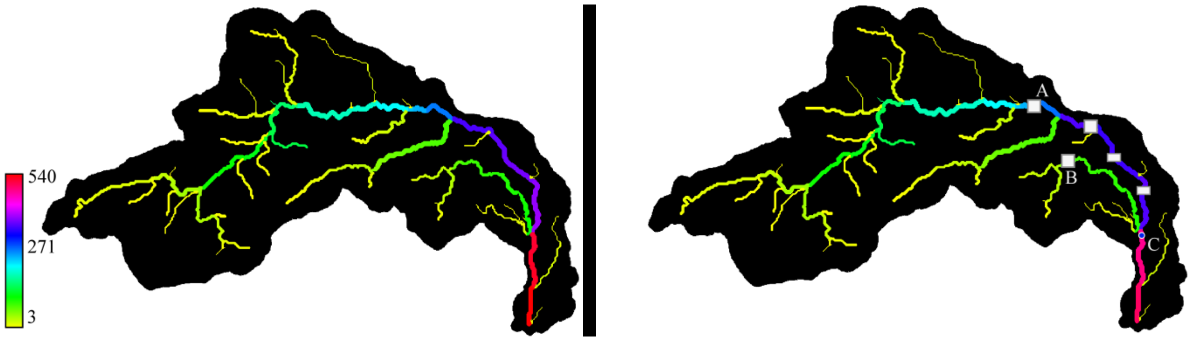

(20) 7 day maximum flow (m^3 s^{-1})

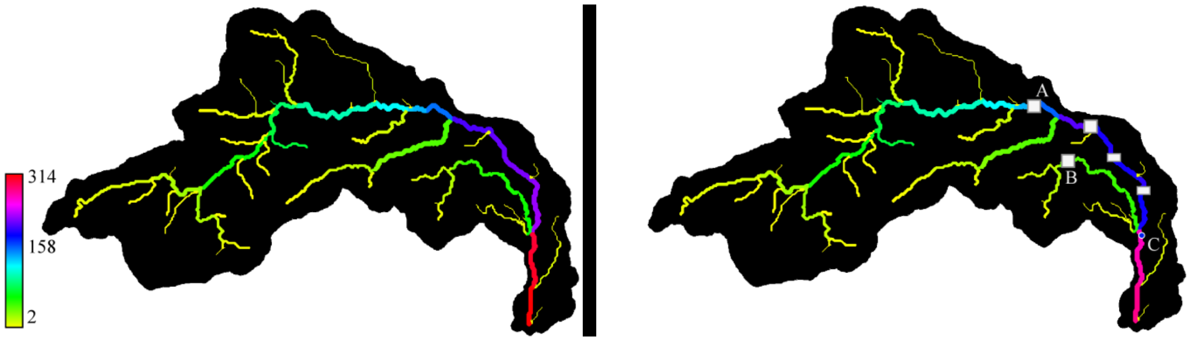

(21) 30 day maximum flow ($m^3\ s^{-1}$)

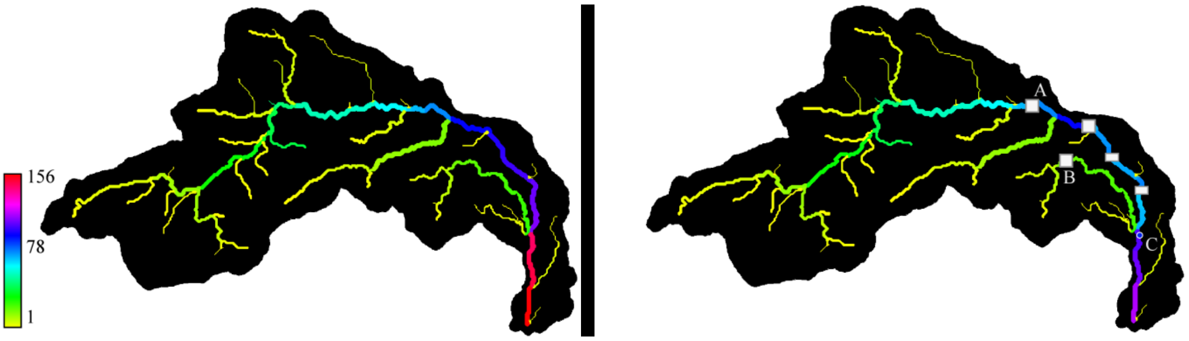

(22) 90 day maximum flow ($m^3\ s^{-1}$)

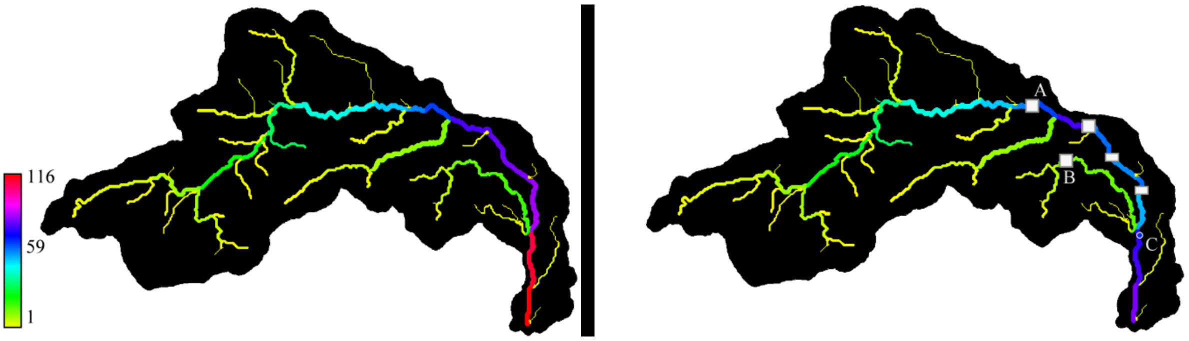

(23) Base flow index

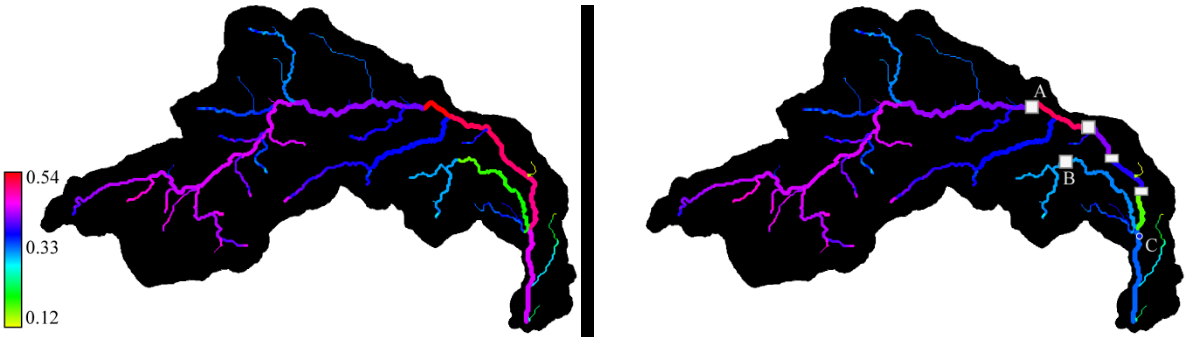

(24) Timing of minimum flow (Julian day)

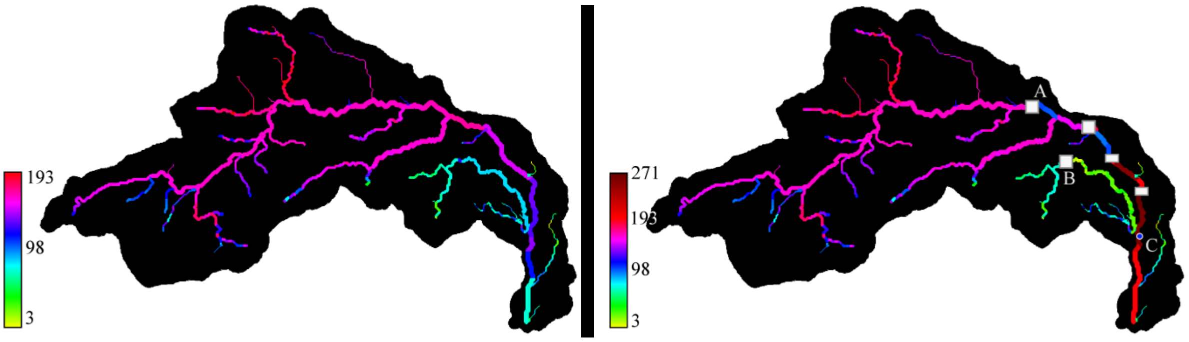

(25) Timing of maximum flow (Julian day)

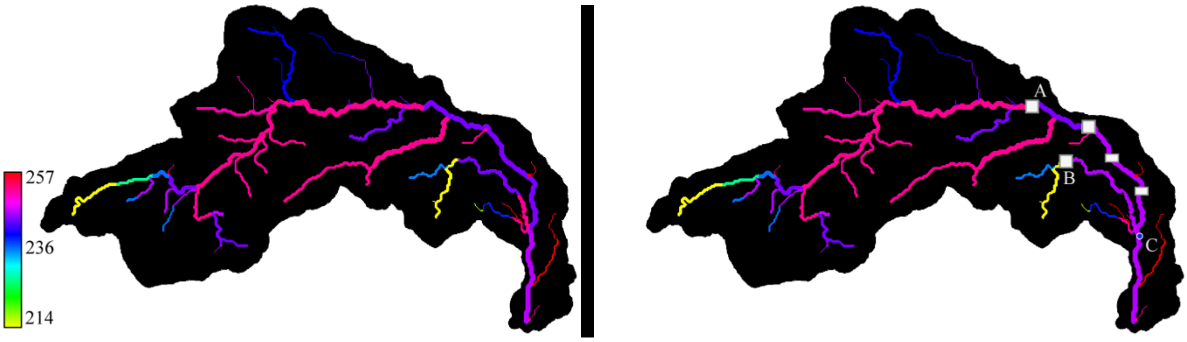

(26) Frequency of low pulse (times year^{-1})

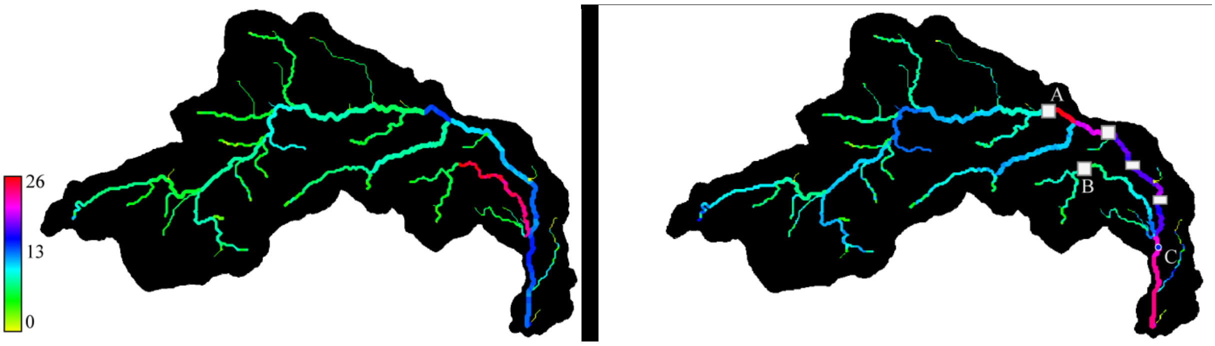

(27) Frequency of high pulse (times year^{-1})

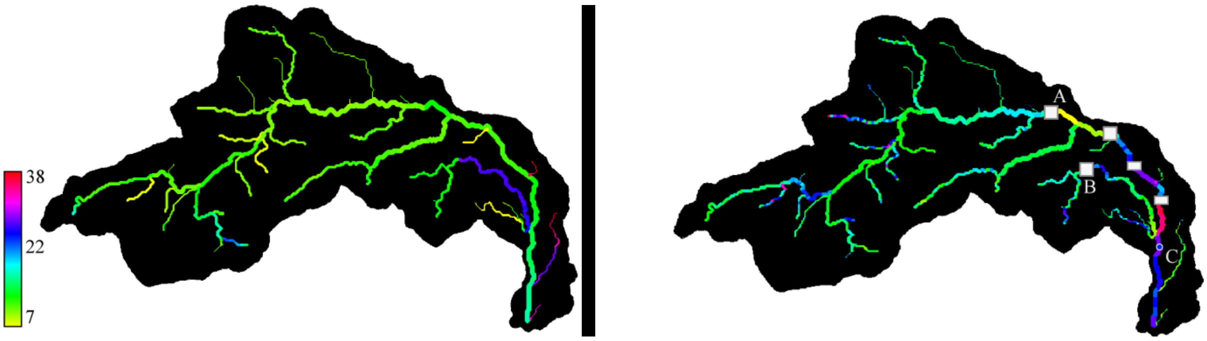

(28) Duration of low pulse (days)

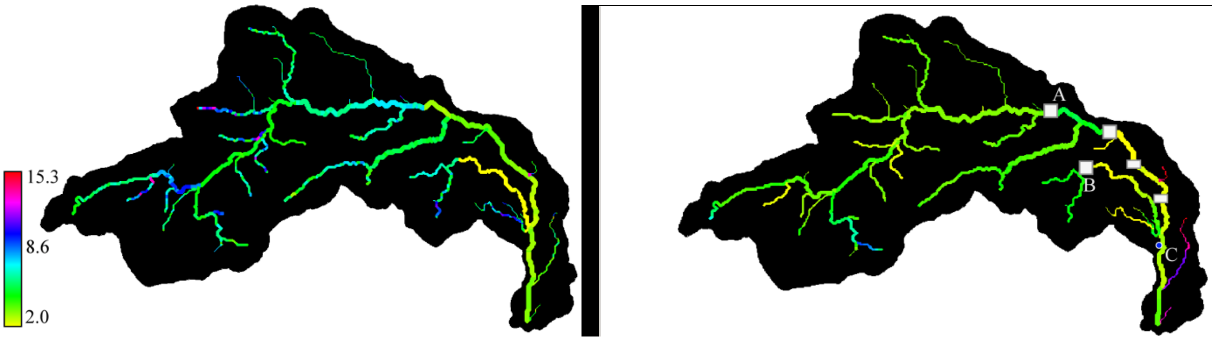

(29) Duration of high pulse (days)

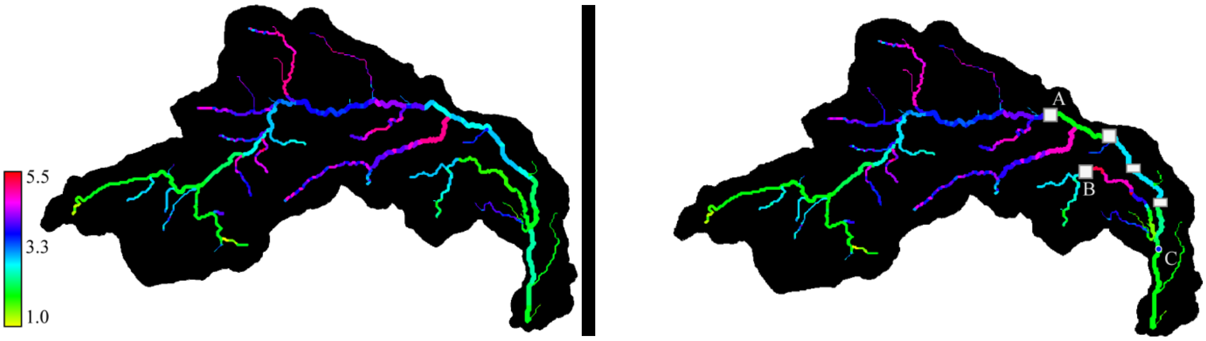

(30) Rise rate (%)

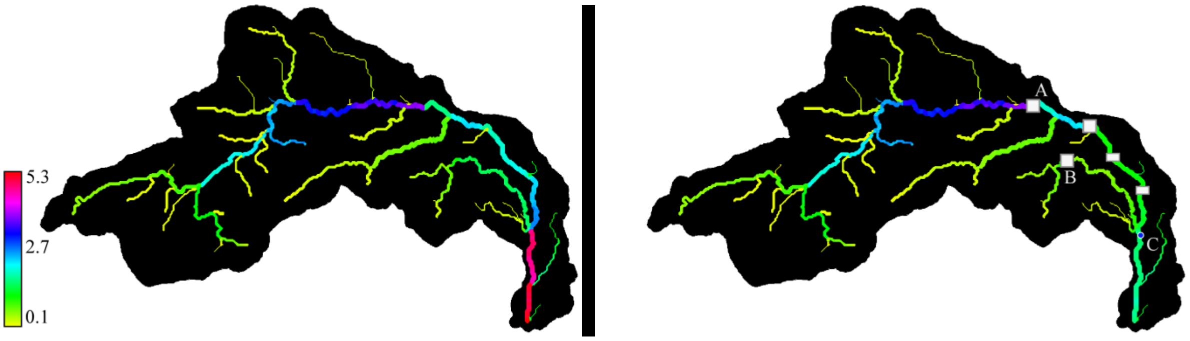

(31) Fall rate (%)

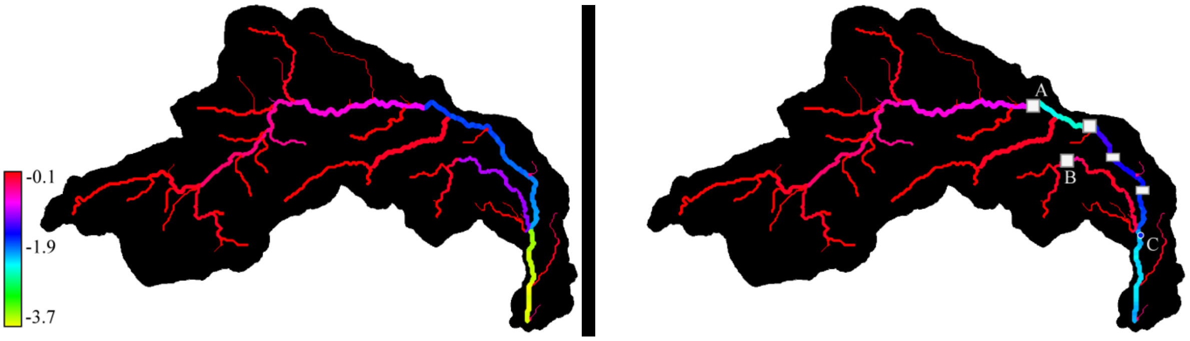

(32) Reversals (times)

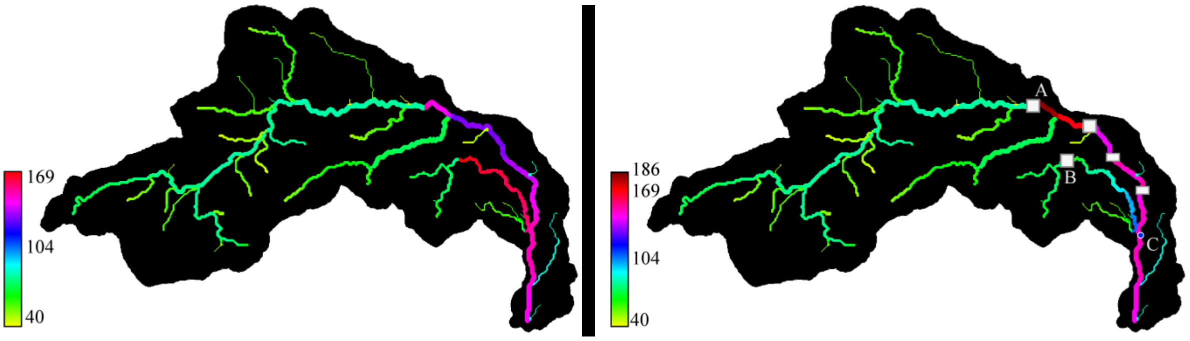

Supplement: S4 Fig — (PDF) [file pone.0133833.s004.pdf]
