# Supplementary material for: Evaluation of Spatial Pattern of Altered Flow Regimes on a River Network Using a Distributed Hydrological Model
Source: PLoS One. 2015 Jul 24;10(7):e0133833. doi: 10.1371/journal.pone.0133833 (PMC4514816; doi:10.1371/journal.pone.0133833)
Supplement: S1 File — (PDF) [file pone.0133833.s005.pdf]

Appendix D. Spatial correlation analysis for the degrees of alterations of (a) median flow in August and (b) frequency of high flow pulses (cf. Fig. 6)

As an example for more detailed analyses, we evaluated correlations of the degree of alteration (DoA) along the river network. We divided the Sagami River and Nakatsu River into six sections A–F (Fig. D1). At each section, the uppermost and lowermost sites (see the example for section A in Fig. D1) were selected for the correlation analysis (i.e., a total of 12 evaluation sites). At each evaluation site, the DoA was calculated for median flow in August and the frequency of high flow pulses each year based on 10-year simulated discharges. The spatial tendencies of the DoAs were shown in Fig. D2.

Correlation coefficients were generally high ($r > 0.7$) within same sections for both hydrologic indices (Fig. D3). Correlation analysis can be also used to evaluate the similarity in the DoAs among sections. For example, the DoAs of monthly median flow in August (Fig. D3 (a)) were similar among the four sections (B, C, D, and F; $r > 0.67$). On the other hand, these sections had low similarity with the other two sections (A and E; $r < 0.4$). Therefore, this result indicates that the inter-annual trend of the DoA was longitudinally similar from section B to F, but the sections A and E experienced different trends of the alteration.

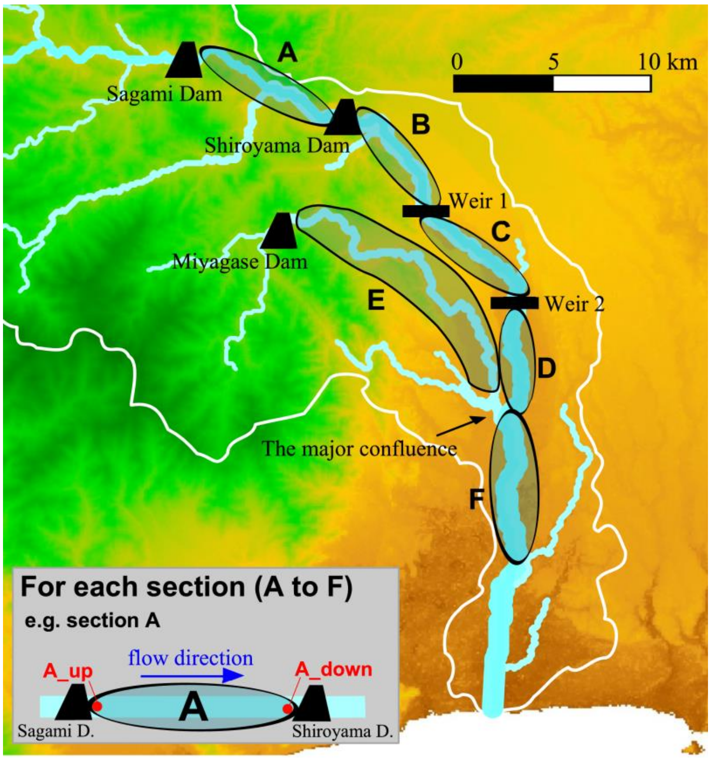

Fig. D1. Six sections assigned along the Sagami River.

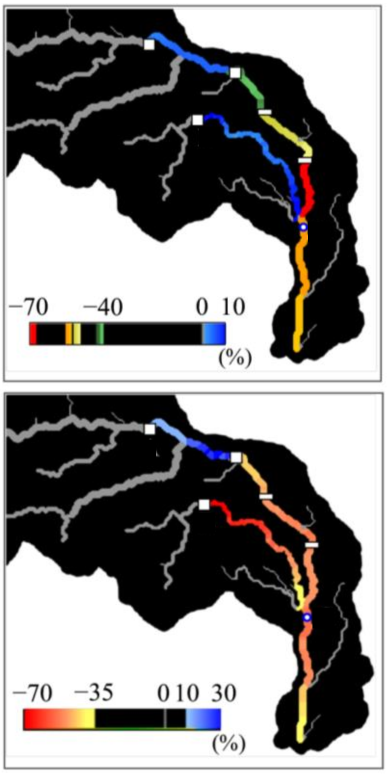

Fig. D2. The degrees of alterations of median flow in August (upper) and the frequency of high flow pulses (lower). Values were caluclated by averaging the DoAs for 10 years (2000–2009). Note that these panels are also shown in **Fig. 6**.

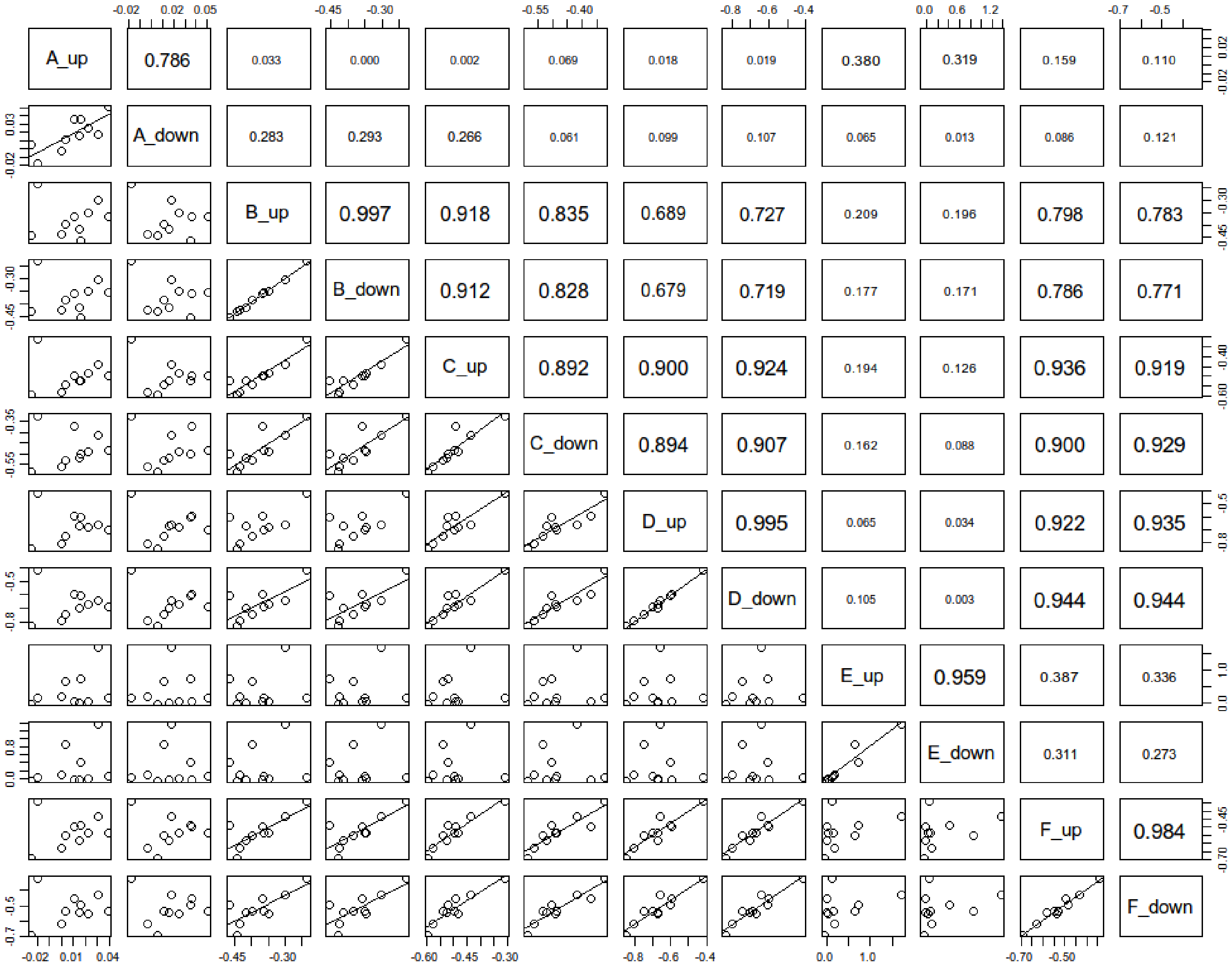

Fig. D3 (a). Correlation matrix for the degree of alteration of median flow in August. The names of evaluation sites are on the diagonal. The *ij* th and *ji* th panels contain Pearson's correlation coefficient and the corresponding scatter plot, respectively, of the *ii* th and *jj* th panels. For instance, correlation coefficient of section A_up (panel (1,1)) and A_down (panel (2,2)) is 0.786 (panel (1,2)), and the corresponding scatter plot is shown in panel (2,1). Regression line is added if r > 0.7.

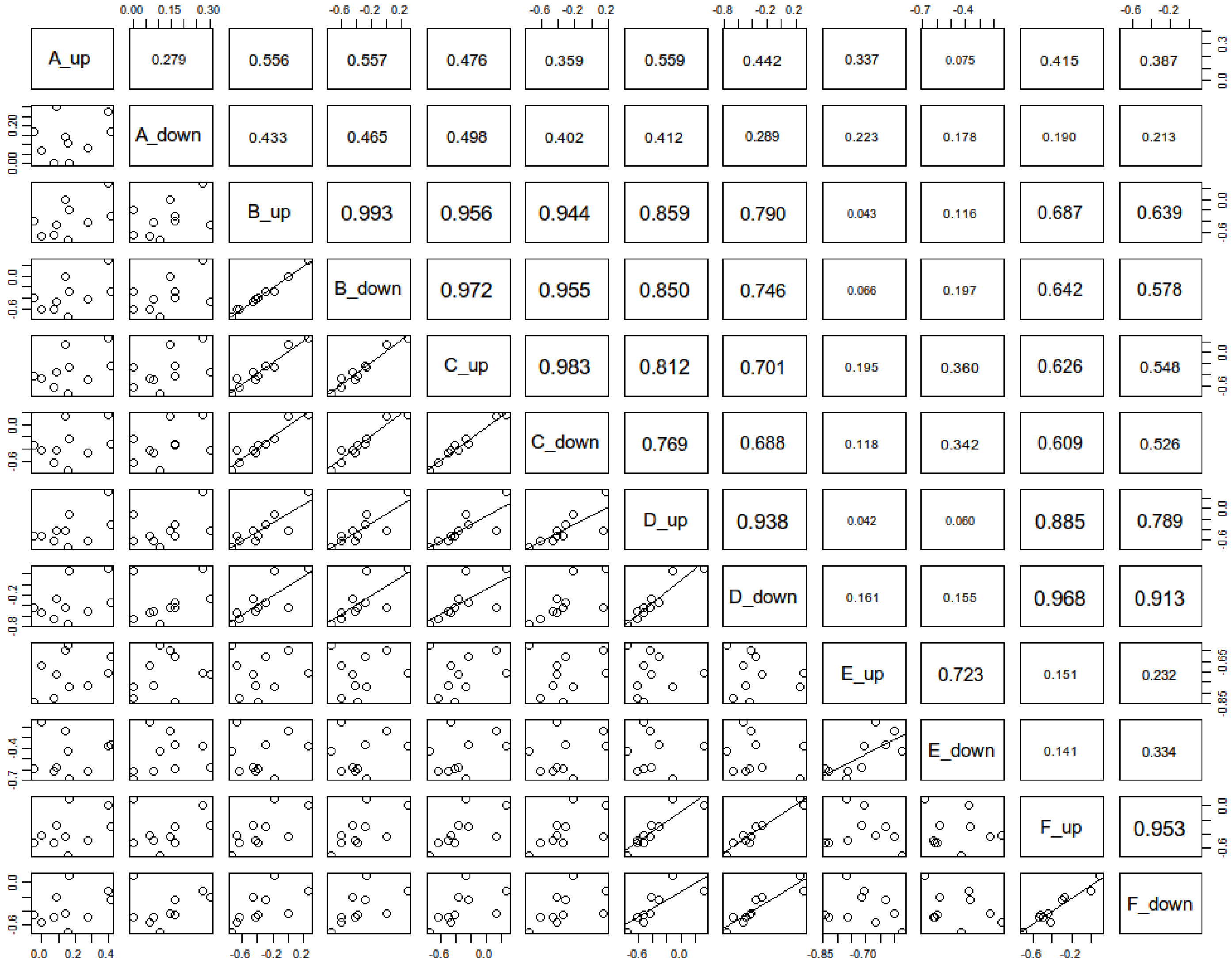

Fig. D3 (b). Correlation matrix for the degree of alteration of frequency of high flow pulses.
