# Supplementary material for: Evaluation of Spatial Pattern of Altered Flow Regimes on a River Network Using a Distributed Hydrological Model
Source: PLoS One. 2015 Jul 24;10(7):e0133833. doi: 10.1371/journal.pone.0133833 (PMC4514816; doi:10.1371/journal.pone.0133833)
Supplement: S1 Table — (PDF) [file pone.0133833.s006.pdf]

(a) Parameters calibrated for landuse type (S1 Fig. a)

Class	Category	Coverage (km^2)	%	Vegetation coverage	NDVI maximum	Crop coefficient	Root depth (m)	Anitropic ratio	Surface storage (mm)	Manning's roughness
1	Water	34	2.0	0	0.1	0.01	0.01	1	0	0.02
2	Urban	155	9.1	0.1	0.5	0.01	0.01	3	1	0.03
3	Paddy	5	0.30	0.5	0.8	0.5	0.3	18	30	0.05
4	Crop	22	1.3	0.7	0.8	0.5	0.3	18	20	0.05
5	Grass	22	1.3	0.8	0.8	0.5	0.7	18	6	0.05
6	Deciduous forest	698	41	0.8	0.8	0.8	4	18	12	0.3
8	Evergreen forest	696	41	0.8	0.8	0.8	4	18	18	0.3
10	Bare land	80	4.7	0	0.2	0.01	0.01	18	6	0.05
	Weighted mean			0.68	0.73	0.67	3.3	16	13	0.25

Note that classes 7 and 9 are not assigned to any classification.

(b) Parameters calibrated for soil type (S1 Fig. b)

Category	Coverage (km^2)	%	Soil depth (m)	Saturated volumetric water content	Residual volumetric water content	Van Genuchten's alpha	Van Genuchten's *n*	Saturated hydraulic conductivity at surface layer ($mm\ hr^{-1}$)	Decay factor *f*	Hydraulic conductivity at ground ($mm\ hr^{-1}$)	Ground water storage coefficient
Sedimentary rock	428	25	4	0.44	0.15	0.083	1.72	338	0.98	0.003	0.15
Volcanic rock	873	51	4	0.45	0.20	0.083	1.90	767	0.98	0.074	0.15
Accretionary complex	411	24	4	0.45	0.20	0.083	1.72	476	0.98	0.046	0.15
Weighted mean			4	0.45	0.19	0.082	1.81	590	0.98	0.049	0.15
